# Parent–child couples display shared neural fingerprints while listening to stories

**DOI:** 10.1038/s41598-024-53518-x

**Published:** 2024-02-04

**Authors:** Nir Habouba, Ronen Talmon, Dror Kraus, Rola Farah, Alan Apter, Tamar Steinberg, Rupa Radhakrishnan, Daniel Barazany, Tzipi Horowitz-Kraus

**Affiliations:** 1 Educational Neuroimaging Group, Faculty of Biomedical Engineering, Faculty of Education in Science and Technology, Technion - Israel Institute of Technology, Haifa, Israel; 2 Faculty of Electrical and Computer Engineering, Technion - Israel Institute of Technology, Haifa, Israel; 3 The Institute of Child Neurology, Schneider Children’s Medical Center of Israel, Petach Tikvah, Israel; 4 The Department of Psychological Medicine, Schneider Children’s Medical Center of Israel, Petach Tikvah, Israel; 5 School of Medicine, Indiana University, Indianapolis, IN USA; 6 The Alfredo Federico Strauss Center for Computational Neuroimaging, Tel Aviv University, Tel Aviv, Israel; 7 Department of Neuropsychology, Center for Neurodevelopmental and Imaging Research (CNIR), Kennedy Krieger Institute, Baltimore, MD USA; 8 Department of Psychology and Behavioral Sciences, Johns Hopkins University School of Medicine, Baltimore, MD USA

**Keywords:** Human behaviour, Biomedical engineering

## Abstract

Neural fingerprinting is a method to identify individuals from a group of people. Here, we established a new connectome-based identification model and used diffusion maps to show that biological parent–child couples share functional connectivity patterns while listening to stories. These shared fingerprints enabled the identification of children and their biological parents from a group of parents and children. Functional patterns were evident in both cognitive and sensory brain networks. Defining “typical” shared biological parent–child brain patterns may enable predicting or even preventing impaired parent–child connections that develop due to genetic or environmental causes. Finally, we argue that the proposed framework opens new opportunities to link similarities in connectivity patterns to behavioral, psychological, and medical phenomena among other populations. To our knowledge, this is the first study to reveal the neural fingerprint that represents distinct biological parent–child couples.

## Introduction

During a child’s development, the caregiver or parent is the most important individual to the child. Behavioral and imaging studies have demonstrated the critical positive^1–6^ and potential negative impact^7,8^ of parent–child interaction on the well-being and cognitive ability of a developing child. Studies suggest that parent–child interaction is related to the development of the child’s limbic^9^ and cognitive (i.e. executive functions^10–12^) systems. These studies point to parental emotional regulation during interaction, modeling to the child and setting an emotional climate at home as key factors facilitating their child's emotional regulation (for review, see^9^). This emotional regulation, in turn, was found to engage neural systems related to executive functions and attention orienting in the child^9^.

A number of neuroimaging studies have expanded our knowledge of the parent–child interaction by demonstrating its associated neurobiological correlates^13–17^. A proposed neurobiological model for parent–child interaction suggests that both cognitive (prefrontal inhibitory circuits, i.e. the dorsolateral prefrontal cortex and the anterior cingulate cortex) as well as limbic (anterior insula and amygdala) brain regions in the parent correspond during emotional regulation behavior and directly affect the activation of corresponding brain regions in the child^9^ (also supported by^18^). This implies that parents tune their children’s brain activity towards the execution of high-level cognitive and social processes^15^. Defining neurobiological correlates for parent–child interaction is possible using hyperscanning methods, enabling parent–child synchronization of brain activity during interaction or while exposed to a similar stimulus measured simultaneously (for review, see^19^). Moreover, similar studies demonstrated that parents and children who were more emotionally synchronized introduced similar resting-state connectivity profiles while watching an emotional movie^16^ as well as during rest^16^. Others examined the neural similarity of mothers observing their teenagers performing a stressful task in the scanner, where greater neural similarity was observed in dyads reporting higher family connectedness^17^.

The Connectome-based Prediction Model (CPM) is another method contributing to the study of within-group variability in the brain-behavior relationship^20,21^. It is a data-driven linear model that was developed for the prediction of behavioral measures from intra-brain connectivity data. The model utilizes cross-validation to overcome the overfitting problem typical for ordinary correlation or regression. Thus, it obviates the need for correction for multiple comparisons and enables a reliable generalization. Recently, the CPM has been found to be a reliable predictor of cognitive abilities, such as fluid intelligence^20,22^.

Another significant contribution to the research of individual variability was provided in the connectome fingerprinting study^20^. This study demonstrated how FC patterns act as a robust and reproducible fingerprint that differentiates individuals by their brain connectivity patterns. More specifically, using the Human Connectome Project (HCP)^23^, researchers showed how intra-brain FC patterns collected from 126 subjects on a first scanning day could be used to accurately predict subject profiles on a second scanning day. The identification was successful within and between sessions and also across resting state and task conditions, including working memory, motor task, language, and emotion-related tasks^23,24^. These results indicated that although brain connectivity patterns change to some degree with the specific demands of a given task, it hides a unique and robust functional organization that serves as an individual fingerprint.

Moreover, while the CPM has been proposed as a method to predict cognitive abilities using intra-brain functional connectivity data, the fingerprinting model was introduced as a technique to identify individuals based on their intra-brain functional connectivity, irrespective of scanning condition and cognitive behavior^20,21^. Notably, it has been demonstrated that the networks enabling accurate identification through the fingerprinting model were also the most predictive of cognitive measures when employing the CPM^20,21^. However, while individual brain connectivity profiles were shown to hold functional patterns that serve as unique and robust fingerprints^20^, it is still unknown whether biological parent–child couples also share distinct connectome-based neural fingerprints.

The aim of this study was to examine whether parents and their biological children share distinct functional connectivity patterns that can be identified from a group of parents and children. To this end, 13 Hebrew-speaking children aged 8–12 years (mean age 9.7 ± 1.3 years, six females) and one of each of their biological parents (mean age 42.4 ± 5.5 years, 11 females and 2 males) were scanned while listening to stories. This task was previously found valuable in revealing brain mechanisms that underlie cognitive abilities and behavioral similarities^6,14,25–27^.

We hypothesized that biological parent–child couples share distinct functional connectivity patterns, mainly those associated with emotional and executive functions systems, and that these patterns would enable identifying couples from brain connectivity profiles acquired in a story-listening task. We also hypothesized that utilizing a diffusion map (DM) framework, a non-linear dimension reduction technique that deals with the multiple dimensionality challenge in functional MRI data, can establish a meaningful representation of parent–child functional-connectivity similarities^28–31^. We also suggest that by utilizing ideas from the connectome-based predictive model and the fingerprint model, intra-brain functional connectivity profiles^32^ obtained from parents and children while listening to stories will enable the identification of their biological child or parent from a set of connectivity profiles. These patterns will be distributed over both cognitive, limbic and sensory brain networks. Furthermore, the CBI model would be specifically designed to predict biological parent–child couples through a-priori examination of the relations between the two investigated groups (biological dyads). Hence, we hypothesized that the embedded a-priori information about the functional relationship between parents and biological children would lead to higher prediction rates when compared to the fingerprinting model.

we propose that the CBI model will outperform the ‘fingerprinting’ model^20^ in predicting biological parent–child couples.

## Materials and methods

### Participants

Thirteen Hebrew-speaking children aged 8–12 years (mean age 9.7 ± 1.3 years, six females) and one of each of their biological parents (mean age 42.4 ± 5.5 years, 11 females) participated in the study. They all had normal hearing and normal or corrected-to-normal vision in both eyes. None of them had contraindications to functional magnetic resonance imaging. All the parents and children were Caucasian, and from an above-average socioeconomic background. Their verbal and non-verbal IQ, as measured by the vocabulary and matrix reasoning tests, respectively^33,34^, were both in the normal range.

Upon enrollment, the parents reported the absence of a history of mental health, and of neurological or developmental disorders for both themselves and their children. The adult participants were recruited through online ads and commercial advertisements. They all signed written informed consent forms confirming their participation and their children’s participation. Children older than ten years also signed assents confirming their participation. The participants were compensated for their time and travel. Schneider’s Children's Hospital Medical Center and the Israeli Ministry of Health Review Board approved the study. All methods were performed in accordance with the relevant guidelines and regulations.

### Procedures

Data were collected in the Alfredo Federico Strauss Center for computational neuroimaging located at Tel-Aviv University (Israel) in two separate sessions for parents and children with a maximal interval of one month between the two scans. Each session included a behavioral assessment and a magnetic resonance scan. The behavioral assessment was administered individually in one of the testing rooms in the neuroimaging center.

In the first session, a set of behavioral tests was administered to assess children’s general aptitude. Then, children were invited to the fMRI room, where they underwent a desensitization procedure^35,36^, explored their environment, and were encouraged to ask questions about the scanning procedure. Children were told that while being scanned, stories would be played and that they should listen carefully so as to be able to answer questions relating to the plots. After fMRI data acquisition was completed, children were tested on the stories. Importantly, as part of the behavioral battery, parents completed a set of questionnaires that assessed their own and their children’s cognitive and behavioral patterns^37–39^.

**Figure 1:**
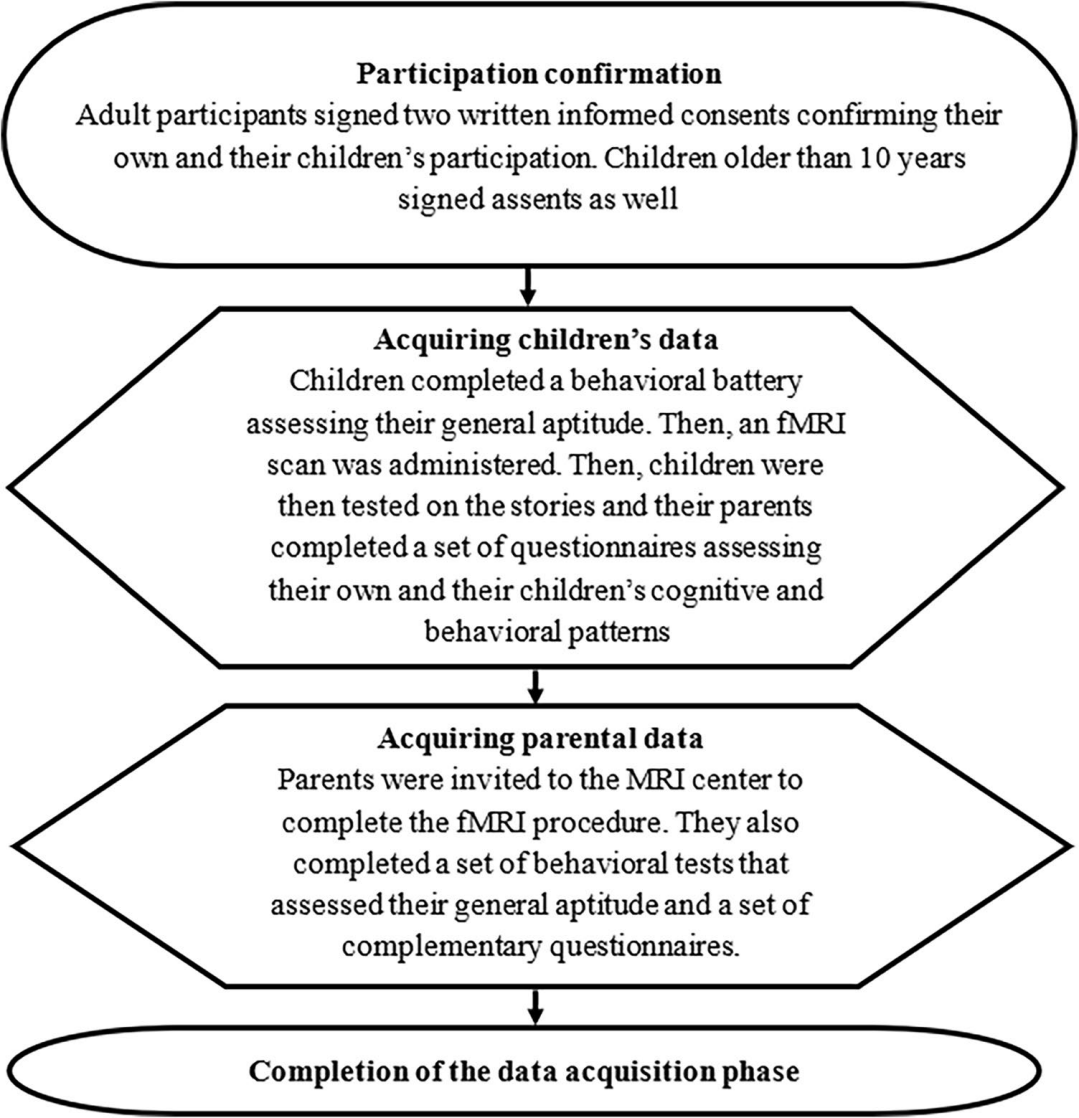
Data acquisition scheme. Both parents and children completed an fMRI scan and cognitive and behavioral assessment.

As part of the second session, the parents were invited to the MRI center to complete an fMRI procedure. Before the scan, a set of behavioral tests was administered to assess general aptitude. Then, parents were told that the same set of stories their children were listening to would be played and that they should listen to the stories so they would be able to answer questions relating to the plots. After completing a desensitization procedure, the experimenter started the scan. Once fMRI data acquisition was completed, and a complementary set of behavioral questionnaires was administered. The full study procedure is described in Fig. 1.

### Behavioral measures

Both adults and children underwent behavioral and cognitive examinations to evaluate aspects of nonverbal and verbal abilities, attention skills, and executive functions. Behavioral data acquisition for both parents and children lasted about two hours.

### Parental behavioral measures

To assess parental cognitive and behavioral skills, a number of tests and questionnaires were administered, as described below.

General verbal and non-verbal abilities, including perceptual reasoning, were assessed using a vocabulary test. Non-verbal skills were evaluated by the matrix reasoning test. Tests were provided by the Wechsler Adult Intelligence Scale iii (WAIS)^33^.

Executive functions were assessed using the Behavior Rating Inventory of Executive Function (BRIEF) self-report questionnaire^37,38^. The questions evaluated inhibition, shifting, emotional control, working memory, planning, organization, and monitoring skills.

Attention abilities were assessed using the d2 test^40^.

### Children’s behavioral measures

To assess children’s cognitive and behavioral skills, a number of tests and questionnaires were administered, as detailed below.

General verbal and nonverbal abilities were assessed by the expressive vocabulary test and the matrix reasoning test, respectively, as provided by the Wechsler Intelligence Scale for Children (WISC)^34,41^.

Attention skills and hyperactivity among children were assessed by their parents by means of the Conners Rating Scale questionnaire, which assesses the probability that a child has an attention disorder^42^.

Executive functions of the children were evaluated using the BRIEF parental report^37,38^. The questionnaire contained similar categories to those provided in the adults’ BRIEF questionnaire, with appropriate adaptation for children. In addition, children performed the Stroop Color-Word Interference subtest for switching/inhibition^43^.

### Experimental setting

#### Functional magnetic resonance imaging acquisition

Both parents and children were scanned in a 3T Siemens Prisma MRI scanner located in the Alfredo Federico Strauss Center for computational neuroimaging at Tel Aviv University, Israel, using a 64-channel head coil. Anatomical images were acquired using a T1-weighted MPRAGE pulse sequence parcellated into 1 × 1 × 1 mm^3^ voxels. Simultaneous multi-slice (multiband) accelerated Echo-planar-imaging (EPI) pulse sequence^44^ was used to acquire T2* weighted images with the following parameters: TE/TR = 28.4/1000 ms, flip angle = 68°, voxel size = 2 × 2 × 2 mm^3^, multiband factor = 4, and Ipat = 2. Each volume comprised 192 axial slices for adults or 160/192/208 for children, each slice 2 mm in thickness (No gaps). In addition, all the participants underwent a clinical FLAIR scan for neurological screening.

For the children participants, the Vannest et al.^35^ and Kraus et al.^36^ scanning protocols were followed to ensure their safety and comfort during the MRI acquisition. Image acquisition started only after a participant felt comfortable. For adults and children alike, the study coordinator visually supervised the procedure throughout the scan. If a participant exhibited overt movement, the scan was stopped, the participant was asked to stay still, and the protocol was then continued. If the participant requested to stop the scan, it was terminated immediately. The study coordinator and the participant communicated through headphones equipped with a built-in microphone.

#### Stimuli and experimental design

**Figure 2:**
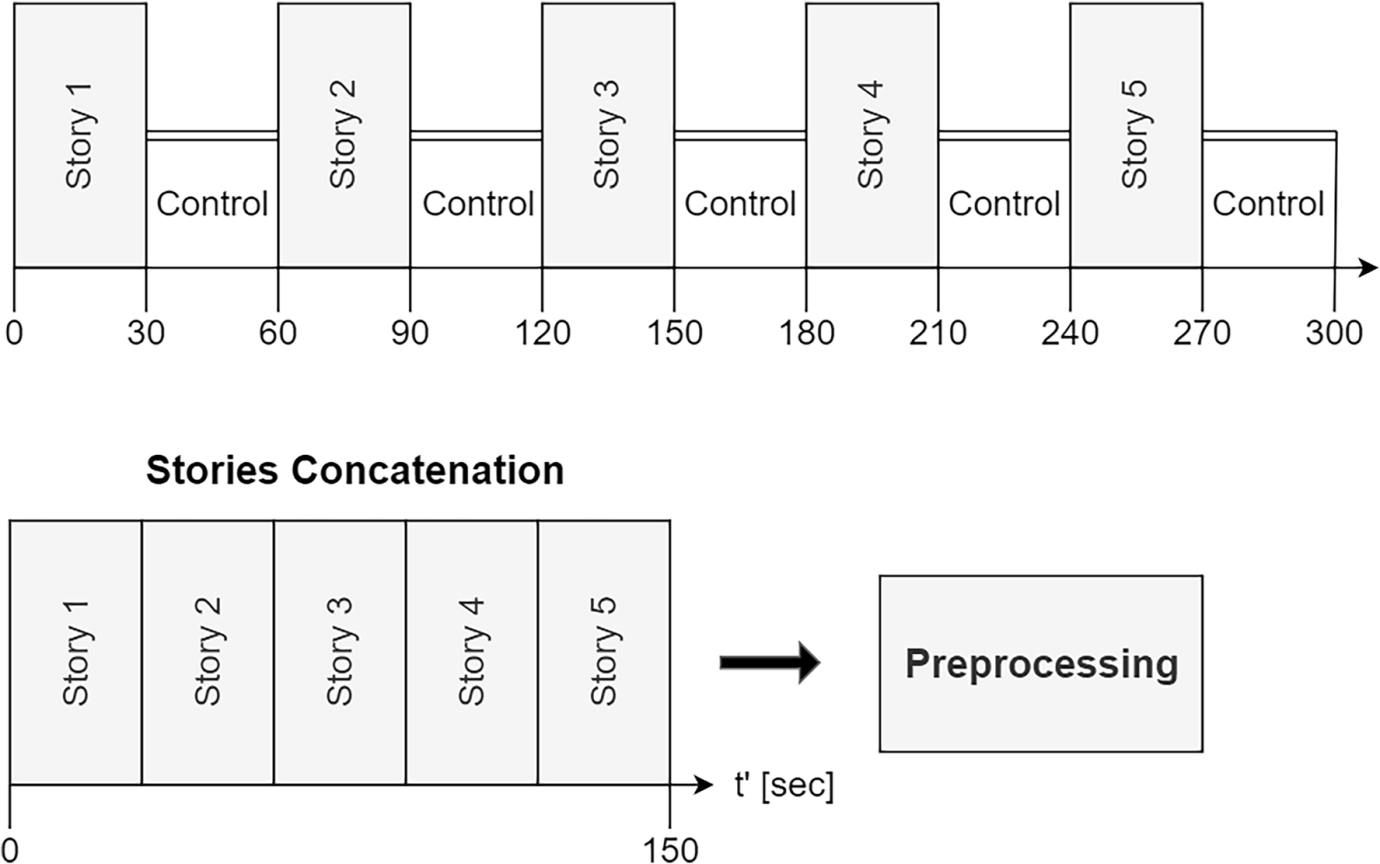
Experimental design. The top of the figure describes the data acquisition design. The bottom section describes how the task blocks were concatenated before preprocessing and data analysis. A similar analysis was also conducted for the control (backward speech) condition.

The participants were scanned while listening to five 30-s-long Hebrew-spoken stories^45^ arranged in a block design (Fig. 2), and read by a neutral female storyteller. The stories were 9–11 sentences in length and consisted of syntactic and vocabulary appropriate for both children and adults and did not include emotional content^45^. The control blocks included backward speech. The child’s attention to the stories was verified using an eye-tracking camera (i.e. verifying they are not sleeping) and by using a listening comprehension questionnaire to ensure a response higher than chance (> 50% accuracy rate), which was administered upon completion of the functional and anatomical scans. Attention to stories in the parents was verified using an eye-tracking camera only.

### Neuroimaging data analyses

#### Functional MRI data preprocessing

Anatomical and functional MRI data were preprocessed in CONN toolbox^32,46^. This included the following steps: slice timing correction, realignment, segmentation, co-registration, normalization, spatial smoothing with a 5 mm FWHM Gaussian kernel^47^, and a framework displacement correction of 0.5 mm. Before preprocessing the five 30-s-long stories, blocks were concatenated^48–50^ (Fig. 2). Using the noise cancellation option in CONN toolbox, temporal frequencies below 0.008 Hz or above 0.09 Hz were removed to minimize the influence of physiological, head motion, and other noise sources such as scanner drift^51^. Functional data were standardized and detrended using the Python NILEARN toolbox^52^. To increase the specificity of the results to the stories condition, the above preprocessing, denoising, standardization, and detrending processes were also applied to the five 30-s-long backward speech control blocks after being concatenated.

#### Regions of interest

**Figure 3:**
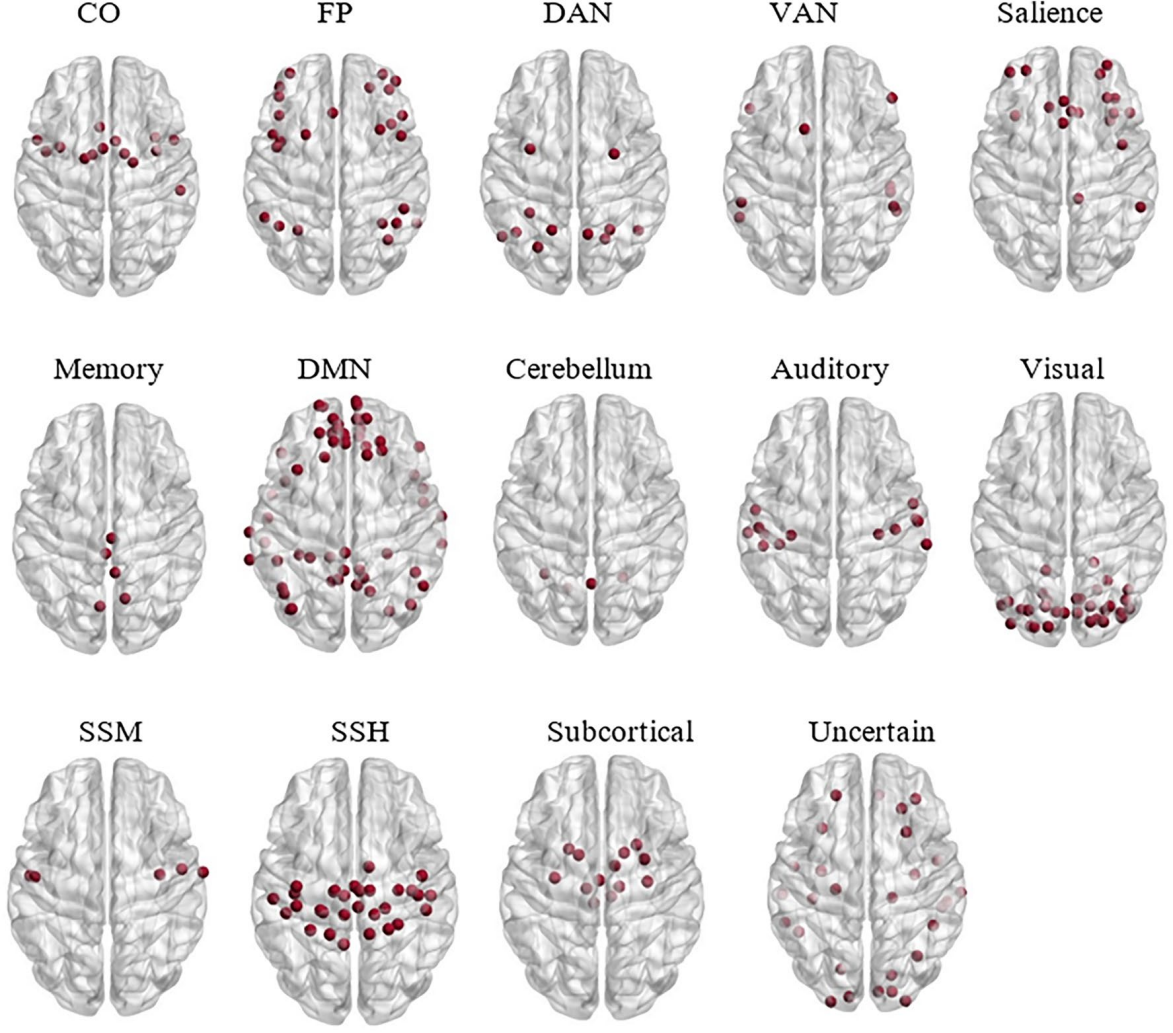
BrainNet Viewer software^54^ visualization of the Power’s brain networks. From left to right, top to bottom, the networks relate to cognitive abilities and somatosensory abilities. The cognitive ability networks are the cingulo-opercular (CO) and fronto-parietal (FP) networks, the dorsal attention network (DAN), the ventral attention network (VAN), salience, memory retrieval, the default-mode network, and the cerebellum. The networks related to somatosensory abilities are the auditory network, the visual network, the somatosensory-mouth (SSM) network, the somatosensory-hand (SSH) network, and the subcortical network. The uncertain network is associated with limbic processing.

**Figure 4:**
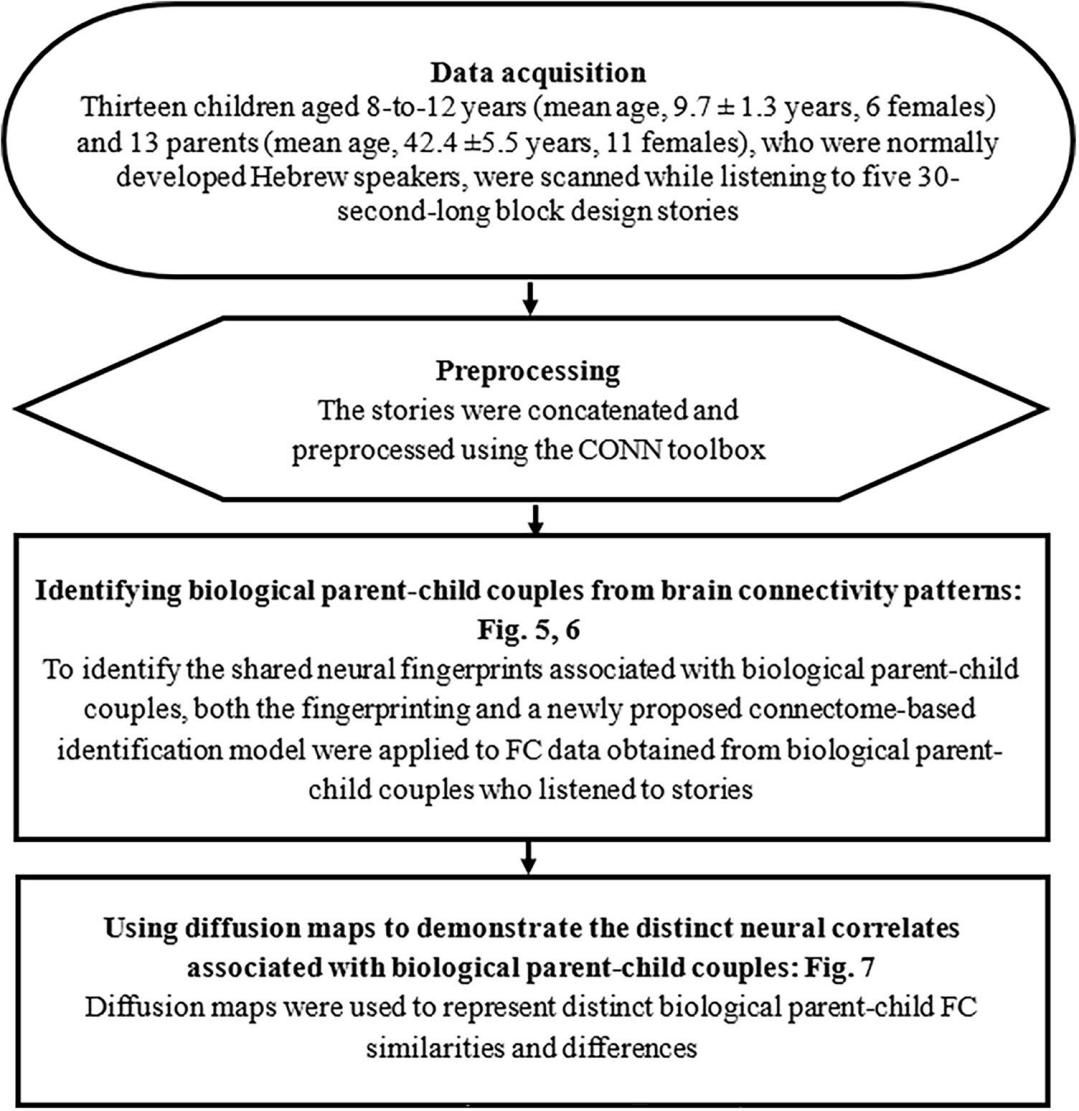
Higher-level analysis pipeline. To examine whether biological parent–child couples share functional similarities. Functional connectivity (FC) similarities were examined using the fingerprinting^20^ model, the connectome-based identification model, and diffusion maps.

For analytical purposes, the Power’s brain parcellation^53^ was applied to the co-registered functional images. This parcellation defines 264 10-mm diameter spherical regions of interest (ROIs), which together constitute 14 functional networks per our hypotheses: eight cognitive networks, five sensory networks, and the uncertain network. The cognitive networks comprised networks related to executive function (FP, cingulo-opercular), attention networks (the dorsal attention network, the ventral attention network, and salience), the cerebellum, the memory, and DMN. The sensory networks comprised visual, auditory, somatosensory-hand, somatosensory-mouth, and subcortical networks. The uncertain network also contained regions associated with limbic processing. This brain parcellation is illustrated in Fig. 3 and the analysis pipeline is noted in Fig. 4. Parcellating the brain into ROIs enabled constructing brain activation profiles that contained 264 rows per all Power’s ROIs and 150 columns corresponding to the sampled data points.

### Postprocessing and higher-level analysi*s*

#### Identification of biological parent–child couples from intra-brain connectivity patterns

To identify biological parent–child couples from patterns of intra-brain functional connectivity, we used the fingerprinting model suggested by Finn et al.^20^ and a new identification model that we call the Connectome-Based Identification (CBI) model. The identification process was applied to FC profiles collected from 13 children and one of each of their biological parents. Each profile was calculated using the Pearson coefficient across the time series of Power’s brain regions^53^. This resulted in an $r x r$ FC symmetric connectivity profile; $r$ represented Power’s 264 brain regions.

#### The Fingerprint Model

**Figure 5:**
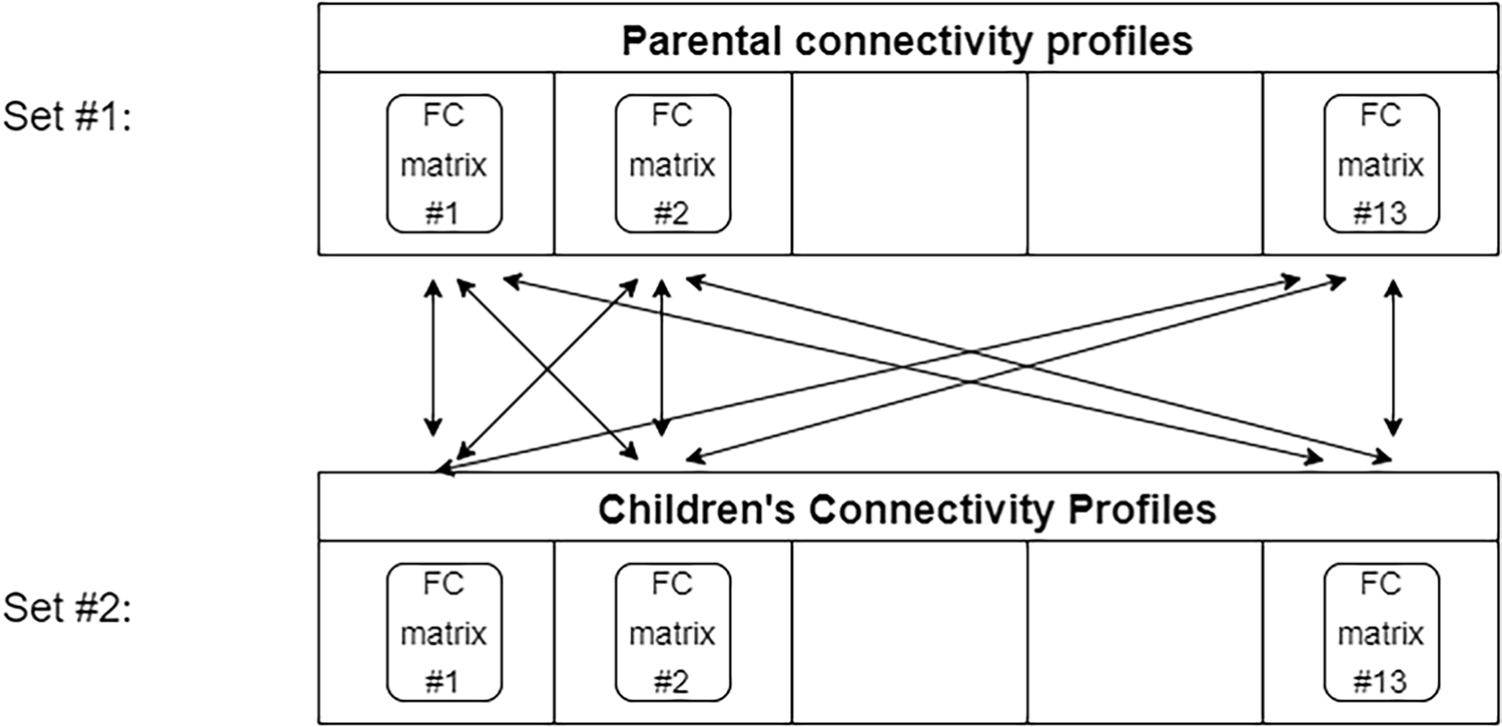
Adaptation of the fingerprint model to identify biological parent–child couples. Thirteen parents and their biological children were assigned to “target” and “database” sets, as suggested by Finn et al.^20^ (denoted here also as set #1 and set #2). The degree of similarity between any two profiles (adult–child) was measured. The arrows indicate all the possible adult–child combinations. The model examined whether biological parent–child couples showed the highest degree of similarity.

To adapt the Finn model^20^ to biological parent–child couples (analogously to the original identification procedure that was developed to identify individuals’ connectivity profiles across two days of separate session), the whole-brain FC profiles of the 26 participating parents and children were assigned to ‘target’ and ‘database’ sets. Each set consisted of 13 parent-only or child-only connectivity profiles. For identification purposes, the parents and their biological children were assigned to the same indices within each set. Then, in an iterative process, a single connectivity profile from the target set was tested against the connectivity profiles in the database set (Fig. 5). The degree of similarity between any two profiles was defined as the Pearson coefficient obtained when correlating their vectorized form. The model was tested by examining whether the most similar connectivity profile of a given parent was his/her biological child. This was considered a successful identification of the biological parent–child couple, and the current iteration was assigned a score of one. Otherwise, the iteration was assigned a score of zero. Then, the overall identification rate was measured by calculating the percentage of couples that were successfully predicted of all the iterations. Subsequently, similar to the fingerprint design, the roles of the two sets were switched and the identification process was repeated.

If the model successfully predicted couples, we evaluated the statistical significance of the results using an a-parametric permutations test whereby the identification procedure was repeated while assigning false identities (indices) to FC profiles. If, for the permutations, the success rates were higher in less than 5% of the iterations ($p < 0.05$), the results were statistically significant. Finally, if the fingerprinting method successfully matched between parents and children, the most prominent network-to-network nodes that were involved in the identification process were explored.

#### The connectome-based identification (CBI) model: an innovative way to identify biological parent–child couples

The CBI model was proposed to improve the identification of biological parent–child couples and to enable an in-depth examination of their interaction. The model was implemented in a number of steps. First, as with the fingerprinting model, the FC profiles of the 13 children and one of each of their biological parents were assigned to two distinct sets: a parental set and a children’s set. Importantly, biological parents and children were assigned the same indices within each set. Then, as with the connectome-based predictive model that correlates brain data with behavioral measures, the two sets were correlated. More specifically, to compare the parental correlation values associated with each ROI-to-ROI connection within the FC profiles and their corresponding children’s values, Spearman’s correlation was performed across sets, for each ROI-to-ROI connection ($r \times r$ connections). This process yielded a $r \times r$ correlation matrix that indicated for each ROI-to-ROI connection the degree of correlation between the two investigated sets. Following the connectome-based prediction model, a significance threshold of 0.05 was determined to select the ROI-to-ROI that were significantly positively or negatively correlated across groups. Per our model, we also proposed to refer to these ROI-to-ROI connections as ‘features’. The positively and negatively correlated features were used to represent each participant (parent/child) within the two sets. Accordingly, for each participant, two vectors (representations) containing his/her own FC values within the selected features were constructed, one for the positive and one for the negative features. Thus, unlike the fingerprinting model and similar to the connectome-based predictive modeling, instead of representing participants by their full FC profiles, they were represented by two separate profile vectors, each containing the correlation values within the features ($p < 0.05$). These features were assumed to contain hidden information about the distinct interaction that was associated with biological parent–child couples.

Next, the positive feature vectors of the children $\overline{p}_{c}$ were assigned to a matrix $P_{c} = \left[ {\overline{{p_{c} }} | c = 1,..,13} \right]$, and the positive feature vectors of the parents $\overline{p}_{p}$ were assigned to a matrix $P_{p} = \left[ {\overline{{p_{p} }} | p = 1,..,13} \right]$. Similarly, the negative feature vectors of the children $\overline{n}_{c}$ were assigned to a matrix $N_{c} = \left[ {\overline{{n_{c} }} | c = 1,..,13} \right],$ whereas the negative feature vectors of the parents $\overline{n}_{p}$ were assigned to a matrix $N_{p} = \left[ {\overline{{n_{p} }} | p = 1,..,13} \right]$. Then, to identify biological parent–child couples, the positive and negative vectors were explored separately.

For the positive features CBI sub-model, the identity of the parent’s child was predicted by calculating both the minimum Euclidean distance and the maximum Spearman’s correlation value between each parent and each of the 13 children. In other words, these were calculated between each parental feature vector within $P_{p}$ and all the children’s vectors within $P_{c}$. The correlation values within a single biological parent–child couple were suggested as being positively correlated when the highest correlation values and the shortest Euclidean distances were maintained, compared to unrelated adult–child couples.

In contrast to the above, for the negative features CBI sub-model, the identity of the parent’s child was predicted by calculating both the maximum Euclidean distance and the minimum correlation value between each parent and each of the 13 children. In other words, these were calculated between each parental feature vector within $N_{p}$ and all the children’s vectors within $N_{c}$. The correlation values between the features’ vectors within each couple were suggested as being negatively correlated when the longest Euclidean distance and the lowest negative correlation coefficient were maintained for biological parent–child couples.

**Figure 6:**
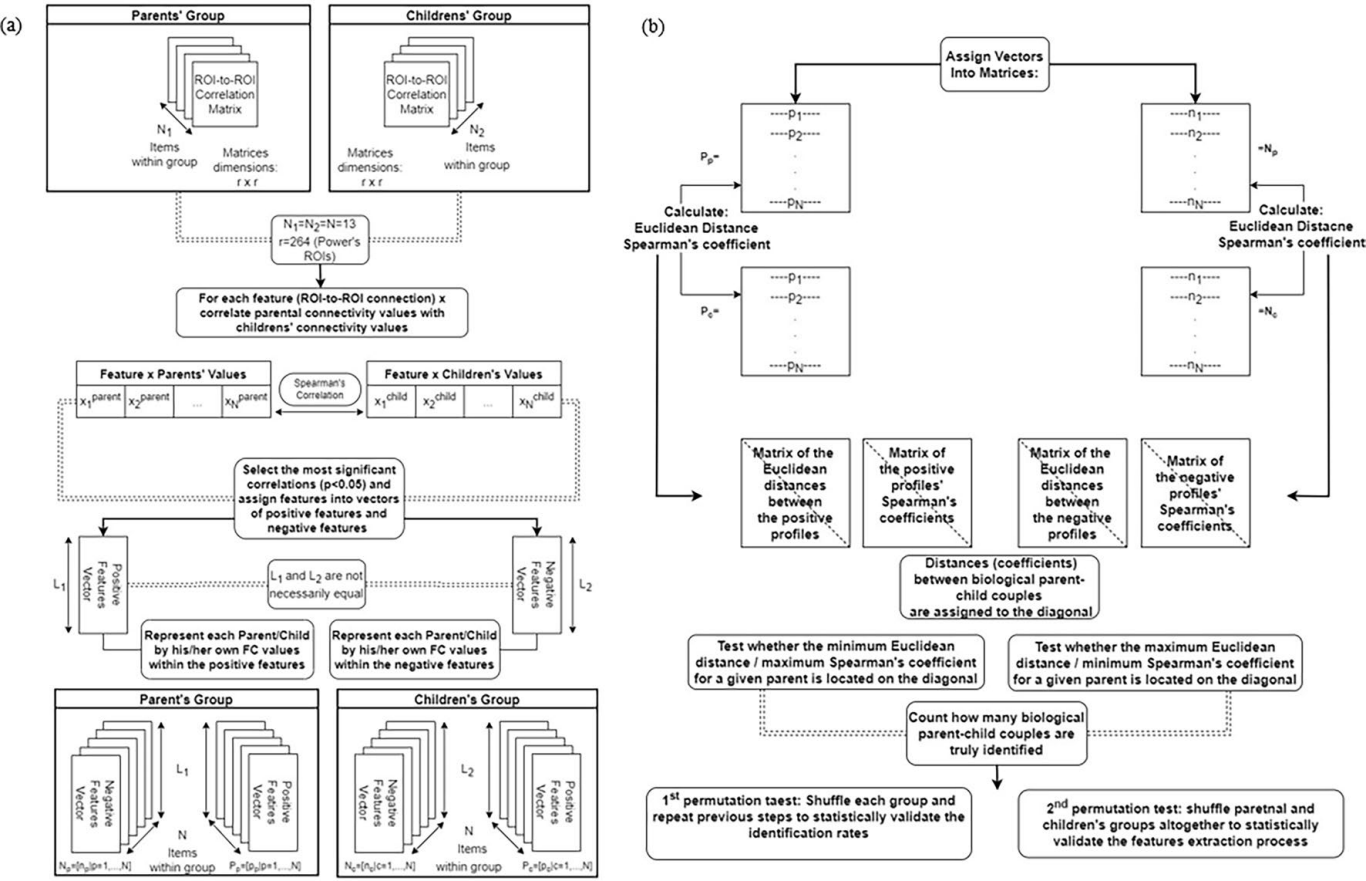
Identifying biological parent–child couples using the connectome-based identification (CBI) model. (**a**) Features extraction: 13 parents and their biological children were assigned to parental and child sets. Then, for each feature, the parents’ and children’s connectivity values were correlated across sets to obtain positively and negatively correlated feature vectors. Within sets, participants were represented by the two vectors: the first vector consisted of functional connectivity (FC) values within the positive features, and the second one consisted of FC values within the negative features. (**b**) Identification of biological parent–child couples: parent and child negative vectors were assigned to matrices N_p_ and N_c_, respectively. Similarly, the positive vectors were assigned to matrices P_p_ and P_c_, respectively. For matrices N_p_ and N_c_, the model tested whether the maximum Euclidean distance (or minimum correlation value) for a given parent was located on the diagonal, thus representing the distance from his/her own child’s profile. For matrices P_p_ and P_c,_ the model tested whether the minimum Euclidean distance (or maximum correlation value) was located on the diagonal. Accurate identifications were those that indicated the maximum and minimum distances on the diagonal that represented parent–child couples per our hypotheses. Finally, two permutation tests were performed to validate the results.

Overall, a total of four ‘distance matrices’ were investigated: two represented the Euclidean distances between $N_{p}$ and $N_{c}$ and also between $P_{p}$ and $P_{c}$, while the other two represented the corresponding Spearman's coefficients. Within matrices, nodes indicated the distances and correlations of a given parent (row) from all the children (columns). As the proposed identification model was based on the assignment of biological parent–child couples to homologous indices within each set, biological parent–child couples were located on the diagonal of the distance matrices. Thus, per the features vector, the model tested whether the maximum Euclidean distance or minimum correlation coefficient (when comparing $N_{p}$ with $N_{c}$), or the minimum distance or maximum coefficient (when comparing $P_{p}$ with $P_{c}$) was located on the diagonal. A parent with successful identification of a biological parent–child couple (i.e., the most similar features vector of a given parent was his/her own child) was assigned a score of one. Otherwise, the parent was assigned a score of zero. The overall identification rate was measured by calculating the percentage of couples that were successfully identified. Figure 6 describes the CBI implementation process.

Finally, to evaluate the statistical significance of the proposed CBI model, two a-parametric permutations tests were conducted. First, an a-parametric 5,000 permutations test was conducted, separately, for the Euclidean-based and the Spearman’s-based results. As the identification model was based on the unshuffled, indices-depending structure of the groups, on each permutation, parents’ and children’s sets were separately shuffled to create false (unrelated) couples. This process ensured that parents and children were assigned the diagonal of the distance matrices only by chance. Thus, in line with the model’s assumption that the maximum and minimum values were located on the diagonal, reflecting a biological parent–child couple, the identification rates were assumed to decrease significantly. This hypothesis was examined by calculating the mean number of true identifications per iteration. Additionally, the percentage of iterations in which the identification rate was equal to or higher than the identification rate calculated before shuffling the data, was measured. This measure was referred to as the model’s prediction rate. A high prediction rate indicated that biological parent–child couples did not necessarily obtain the maximum or minimum distances and coefficients between their negative and positive feature vectors. However, if the overall prediction rate of true couples was smaller than 0.05, the results were considered significant. Importantly, through statistical verification, the CBI model obviated the need to correct for multiple comparisons. Accordingly, a large number of false positives was reflected in high p-values while performing the first permutation test. In the second 5,000 permutations test, on each iteration, the parental and children’s two sets were shuffled. Then, Spearman’s correlation was performed across the two shuffled sets, yielding a $r \times r$ correlation matrix that indicated for each ROI-to-ROI connection the degree of correlation between the two shuffled sets. Following the connectome-based prediction model, a significance threshold of 0.05 was determined to select the ROI-to-ROI that were significantly positively or negatively correlated across the sets (aka ‘features’). The positive and negative features were then applied to the original parent-only and child-only groups to represent each participant (parent/child) by his/her own FC values within the selected randomized features. Then, on each iteration, the identification rates were measured per the new representations. In line with the model assumption that correlating the original sets yielded features containing hidden information about the distinct interaction associated with biological parent–child couples, it was hypothesized that correlating the shuffled groups to obtain features-based representations (positive and negative) would result in a significant decrease in identification rates. This hypothesis was examined by calculating the maximum identification rate and its recurrence. Overall, when compared to the original identification rates obtained for the unshuffled parental and children’s sets, low identification and recurrence rates indicated our first choice of features indeed embedded information about the distinct interaction associated with biological parent–child couples.

Next, if the CBI presented significant and successful identification and prediction rates, another analysis step was done to reveal the network-to-network connections (nodes) that embedded a biological parent–child neural fingerprint, enabling their identification. First, the numbers of positive and negative features constructing the vectorized representations were calculated. However, due to the symmetrical nature of the FC data from which the features were extracted, they also contained duplicate elements that represented the same ROI-to-ROI correlations (i.e., features). To remove these duplicate features, binary symmetric correlation matrices were obtained separately for the positive and negative features of the sub-models, indicating the correlative features. Then, duplicates were removed by counting only the features within the diagonal and the upper (or lower) triangle of the symmetric matrix. More specifically, for the off-diagonal network-to-network nodes, the total number of correlative features was counted, while for the on-diagonal symmetric connections, the number of correlative features within the upper (lower) triangle was calculated. This process yielded two matrices (one for the positive sub-model and one for the negative sub-model), thus indicating the nodes that contributed the highest number of the various features to the identification process. Additionally, for each network-to-network node, the number of positive (negative) features was also normalized for the total number of selected features within the positive and negative vectors. Therefore, two additional matrices were obtained, which indicated the nodes that contributed the largest portion of the various features to the positive and negative identification methods. Subsequently, to identify the nodes that were the most correlated and anticorrelated for biological parent–child couples, the number of the various features was also normalized for node size. This size refers to the number of within-node features for the off-diagonal network-to-network nodes, or the number of features within the upper (or lower) triangle for the on-diagonal nodes. This process contributed one additional matrix for each sub-model, thus illustrating the ‘relative contribution’ of the nodes to the biological parent–child neural fingerprint that enabled successful identifications. Finally, the results obtained for the positive and negative models were compared, and diffusion maps were applied to graphically and quantitively demonstrate the neural similarities and differences associated with the distinct biological parent–child interactions.

#### Exploring the biological parent–child interaction using diffusion maps

**Figure 7:**
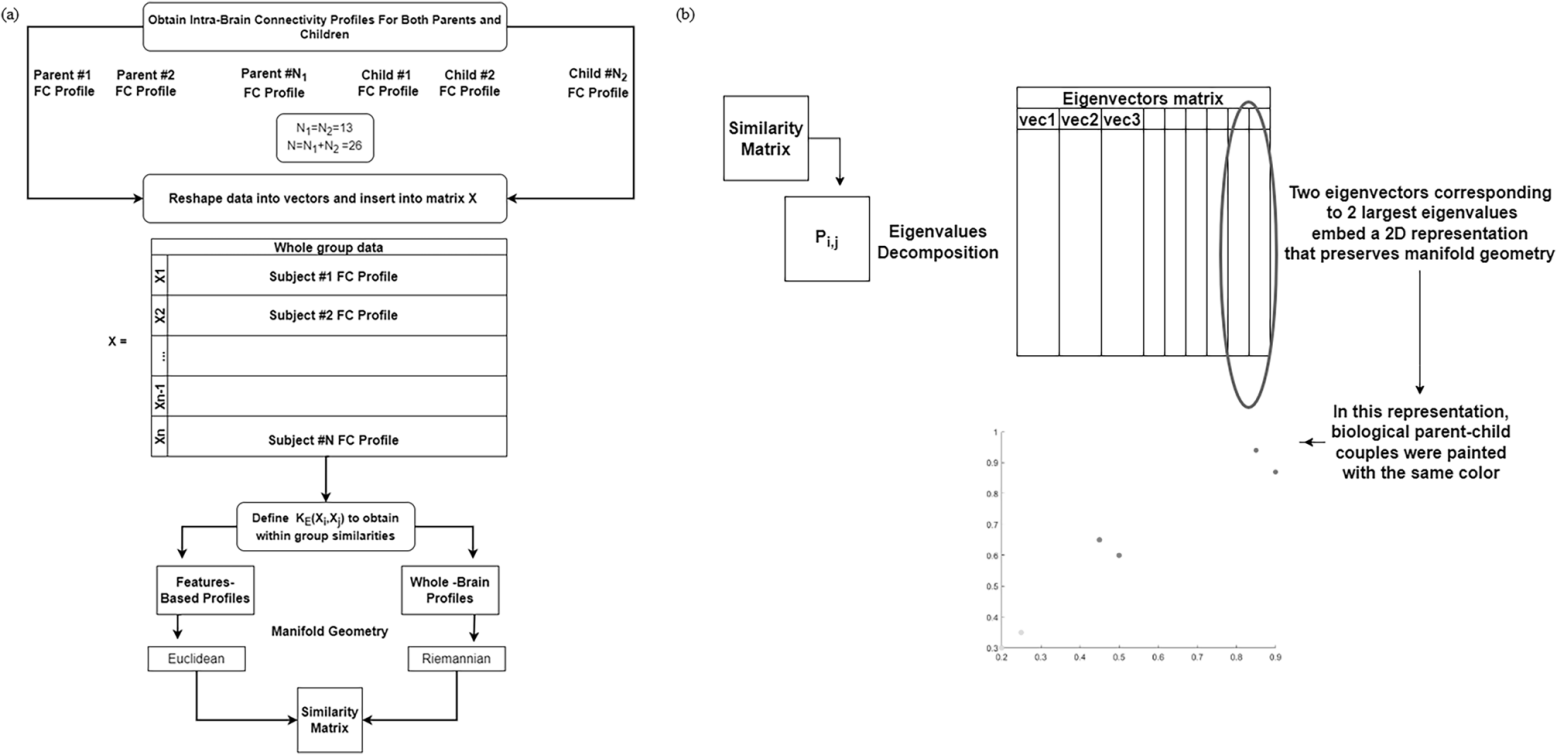
Exploring the biological parent–child relationship with diffusion maps. (**a**) Parents’ and children’s connectivity profiles were flattened into vectors X_i_ to construct a whole group data matrix X containing 26 profiles. Similarities between X_i_s were calculated upon the Riemannian or Euclidean metric and then inserted into a ‘similarity matrix’. (**b**) By using the two eigenvectors corresponding to the two highest eigenvalues of the probability matrix P, a 2D representation was obtained. Biological parent–child couples were shown by the same color.

Aiming to find the neural fingerprint for biological parent–child couples, DMs were constructed for two data sources. The first was the whole-brain FC profiles that were obtained for biological parent-children couples when the fingerprint model was applied. The second was the positive and negative feature profiles that were obtained when the positive and negative CBI sub-models were applied, respectively. Overall, a total of 26 connectivity profiles (features-based or whole brain-based), corresponding to 13 children and one of each of their biological parents, were explored. However, while one map was obtained for the dataset of the whole-brain FC profiles, two distinct maps were constructed for the dataset of the positive and negative feature profiles. One of these maps was for the positive and the other for the negative features-based CBI sub-model. In investigating each dataset, the within-dataset connectivity profiles denoted by $$\left\{ {X_{i} {|}i = 1, \ldots ,N = 26} \right\}$$ were assumed to lie on a low-dimensional sub-manifold *M ,* with density function $$q$$ acting as nodes connected by edges. Each edge represented similarity $$\{ X_{i}$$ and $$X_{j} |\left( {i,j} \right) \in 1,$$…,N}, and was estimated by the following non-negative and symmetric affinity Gaussian kernel: $$K_{\varepsilon } \left( {X_{i} ,X_{j} } \right) = \exp \left\{ { - \frac{{\left| {\left| {X_{i} - X_{j} } \right|} \right|^{2} }}{\varepsilon }} \right\}$$. It was assumed that the low-dimensional representation could be obtained only for sufficiently large ε > 0, which ensured that all the nodes were well connected. Importantly, $$K_{\varepsilon }$$ differed between the two data sources described above. For the data-source of the positive and negative features profiles, the Euclidean norm was applied to calculate the affinity between features-based profiles. In contrast, for the data source whole-brain FC profiles, the Riemannian norm^55^ was applied due to the symmetric and positive nature of these profiles. The probability that a random walker jumped from $$X_{i}$$ to $$X_{j}$$ in a single time step was calculated by normalizing $$K_{\varepsilon }$$ as follows:$$P_{\varepsilon } \left( {X_{i} ,X_{j} } \right) = \frac{{K_{\varepsilon } \left( {X_{i} ,X_{j} } \right)}}{{d_{\varepsilon } \left( {X_{i} } \right)}}; d_{\varepsilon } \left( {X_{i} } \right) = \mathop \sum \limits_{i = 1}^{N} K_{\varepsilon } \left( {X_{i} ,X_{j} } \right)$$. The normalized matrix $$P_{i,j} = P_{\varepsilon } \left( {X_{i} ,X_{j} } \right)$$ represented transitions in a Markov chain with asymptotic behavior. Its eigenvalues decomposition satisfied that: $$1 > \lambda_{0} > \lambda_{1} \ge \lambda_{2} \ge \ldots \ge \lambda_{N - 1} \ge 0$$. By calculating the $$l$$ eigenvectors $$\left\{ {\phi_{i} {|}i = 1, \ldots ,l} \right\}$$ that corresponded to the $$l$$ largest eigenvalues, a $$l$$ -dimensional embedding could be obtained and the profiles were mapped to distinct coordinates. Per our choice, 2D ($$l = 2)$$ represented diffusion maps where $$\left\{ {X_{i} \to \left[ {\lambda_{1} \phi_{1\left( i \right),} \lambda_{2} \phi_{2\left( i \right),} ]} \right.{|}i = 1, \ldots ,N = 26} \right\}$$ were obtained. The nodes, representing 26 profiles of parents and children, and biological parent–child couples were shown in distinct colors (Fig. 7). Additionally, to examine the results obtained for the data source of the positive and negative features profiles, we calculated the mean low-dimensional distances that were associated with biological parent–child couples vs. unrelated couples (i.e., the mean over all possible unrelated couples) and their standard errors. Subsequently, we compared the Euclidean distances of biological couples to those of unrelated couples using an unpaired Mann–Whitney test for populations with unknown distributions, assuming equal medians as the null hypothesis. We report the mean low-dimensional distances associated with biological parent–child couples vs. unrelated couples, their standard errors, as well as the test decisions and its corresponding p-value. Finally, we note that 2-dimensional embedding was used for both visualization and computing distances because it yielded satisfactory empirical results.

## Results

### Baseline characteristics

Thirteen Hebrew-speaking children aged 8–12 years (mean age 9.7 ± 1.3 years, six females) and one of each of their biological parents (mean age 42.4 ± 5.5 years, 11 females and 2 males) were scanned while listening to stories. Table 1 summarizes the cognitive and behavioral data of the participating parents and children. Parental socioeconomic status was above average, including a mean 17.15 (± 1.93) years of education and an above-average monthly income ($5.5–$20 K) according to the Israeli income scale.

**Table 1:** Cognitive measures of the participating parents and children.

Group	Measure	Description (test)	Mean (SD)
Parents	General ability	General nonverbal intelligence (WAIS-III matrix reasoning^33^; standard score)	12.92 (2.53)
		General verbal intelligence (WAIS-III vocabulary^33^; standard score)	10.61 (0.93)
	Attention	Selective and sustained attention (D2-test^40^; concentration performance)	169.29 (25.74)
	Cognitive control	Executive functions abilities (BRIEF questionnaire^37,38^; General score-percentile)	53.45 (25.75)
Children	General ability	General nonverbal intelligence (WISC matrix reasoning^34,41^; standard score)	8.85 (2.97)
		General verbal ability (WISC vocabulary test^56^; standard score)	11.69 (3.30)
	Attention	ADHD screener (Parental Conners questionnaire^42^; probability score)	50.54 (26.56)
	Cognitive control	Executive functions abilities (BRIEF questionnaire^37,38^; General score- percentile)	62.38 (29.10)
		Switching/inhibition test (Stroop color and word test ^43^; Total errors standard score)	10.87 (1.94)

WAIS: Wechsler Adult Intelligence-III; Normal subtest score is 10(± 3).

WISC: Wechsler Intelligence Scale for Children; Normal subtest score is 10(± 3).

BRIEF: Behavior Rating Inventory of Executive Function.

*ADHD* Attention deficit hyperactivity disorder.

### Neuroimaging results

Data for the current study included 26 functional magnetic resonance scans obtained from the participating children and their biological parents. The participants were scanned while listening to five 30-s-long stories in Hebrew^45^, arranged in a block design and read by a female narrator. Before preprocessing, the story blocks were concatenated^48–50^ (see also the online methods). Then, the Power’s brain parcellation^53^ (Supplemental material) was applied to the co-registered functional images. This parcellation defined 264 spherical 10-mm diameter regions of interest (ROIs), which together constituted the 14 functional networks shown in Table 2. ROIs were Pearson-manner correlated to construct 26 symmetrical intra-brain functional connectivity (FC) profiles of 264 × 264 dimensions. Within these profiles, we termed each ROI-to-ROI connection a ‘feature’. For later sensitivity analysis, the five 30-s-long control backward speech blocks were analyzed in the same manner, yielding another 26 control FC profiles. Of note, the average accuracy rate for the narrative comprehension task was above chance (average = 60%, sd = 6%).

**Table 2:** Fourteen functional networks related to cognitive, sensory, and limbic processing were correlated according to Pearson correlations to construct 26 intra-brain functional connectivity profiles corresponding to stories-listening task.

Domain	Network
Cognitive	Fronto parietal* Cingulo opercular* Dorsal attention* Ventral attention* Salience Cerebellum Memory DMN
Sensory	Visual Auditory Somatosensory-hand and mouth Subcortical
Limbic	Regions associated with Power’s uncertain network (supplemental material)^53^

*Networks related to executive function.

*DMN* Default mode network.

### Biological parent–child couples share distinct neural fingerprints

To identify couples of children and their biological parents based on their particular FC profiles, fingerprinting^20^ and the newly proposed CBI models were used. For the fingerprinting model, whole-brain connectivity profiles of all 26 participants were assigned to ‘target’ and ‘database’ sets^20^. Each set consisted of 13 parent-only or child-only FC profiles. Identification rates were measured for the target-database of children-parents and its reverse form, i.e., parents-children. The identification rates were 15.4% (2/13 dyads) and 7.7% (1/13 dyads), respectively. For the control blocks, similarly to the task blocks, the identification rates were 15.4% (2/13 dyads) and 7.7% (1/13 dyads), respectively (similarly to the task condition). In the absence of any specific hypothesis regarding the brain networks that may embed the parent–child shared fingerprinting, these results obviated the need for additional significance tests and indicated that the fingerprinting method failed to identify biological parent–child couples.

Notably, the CBI model enabled more accurate identification of biological parent–child couples. For this model, parental connectivity values that were associated with each ROI-to-ROI connection (or ‘feature’) within the parent profiles were tested against their corresponding values in children. The correlation was estimated using Spearman’s coefficient. Then, features with significant positive and negative correlations across groups (sets) were assigned to corresponding feature vectors. This enabled constructing two representations for each participant (parent and child), consisting of their own correlation values within the features. To identify parent–child couples, the Euclidean distance and Spearman’s coefficient were calculated between parental positive/negative vectors, and each of the children’s positive/negative vectors, respectively. For the Euclidean distances, the identification rates were 100% (13/13 couples) and 92.3% (12/13 couples) for the positive and negative feature-based representations, respectively. For Spearman’s coefficients, the identification rates were 100% (13/13 couples) for both the positive and the negative features. The results were then verified through two permutation tests. In the first test, each set was iteratively shuffled. After shuffling, the mean identification rates for both the Spearman-based and the Euclidean distance-based methods dropped to 15.4% (2/13 couples) and 7.7% (1/13 couples) for the positive and negative features, respectively; a zero non-parametric $p$ value was calculated. For the second permutation test, the two sets of parents and children were shuffled on each iteration. Then, Spearman’s correlation with a significance threshold of 0.05 was performed across the two random sets to iteratively obtain positive and negative features. These features were then used to represent each participant (parent/child) vector-wise within the original parent-only and child-only sets based on their own FC data, and identification rates were measured per the new representations.

Overall, for the Euclidean distances, the maximum identification rate measured was 38.462% (5/13 couples), occurring in 0.0006% (3/5,000) of the permutations for positive features, and 38.462% (5/13 couples) occurring in 0.0002% (1/5,000) of the iterations for negative features. Mean identification rates were 0.798 and 1.082 accurate couples, respectively. For the Spearman’s coefficients, the maximum identification rate measured was 38.462% (5/13 couples), occurring in 0.0004% (2/5,000) of the permutations for positive features, and 46.154% (6/13 couples) occurring in 0.0002% (1/5,000) of the iterations for negative features. Mean identification rates were 0.923 and 1 accurate couples, respectively. These results indicated low identification rates compared to those obtained when identifying couples upon correlation across the unshuffled sets, thus strengthening the results.

**Figure 8:**
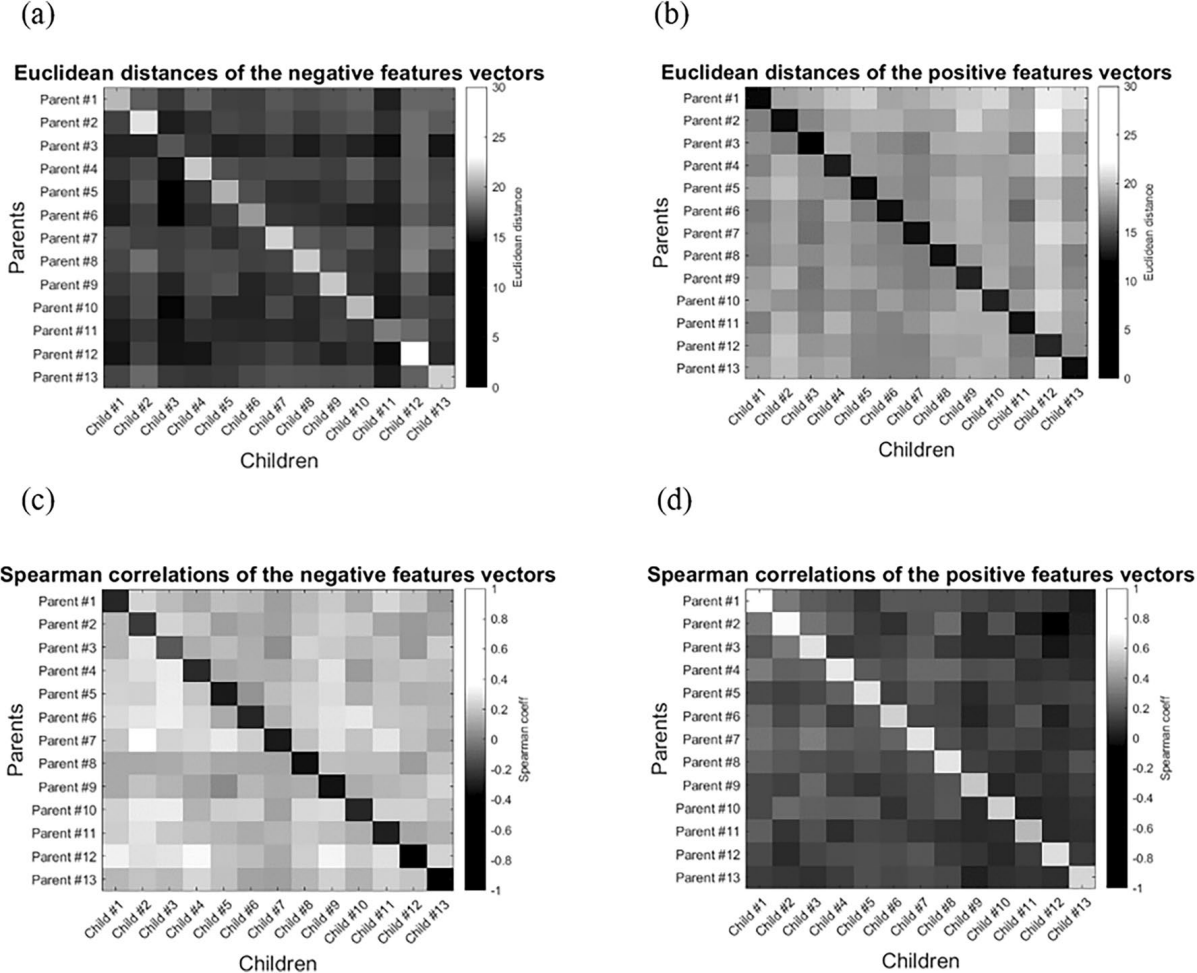
The Connectome-based Identification (CBI) results. (**a**) The Euclidean distance between parents (rows) and children (columns), as calculated for the negative feature vectors (i.e., negative CBI sub-model). The maximum distances were located on the diagonal representing biological parent–child couples. (**b**) The Euclidean distance between the parents (rows) and children (columns), as calculated for the positive feature vectors (i.e., positive CBI sub-model). Minimum distances were located on the diagonal, representing biological parent–child couples. (**c**) Spearman’s correlation coefficients between parents (rows) and children (columns), as calculated for the negative feature vectors (i.e., negative CBI sub-model). Minimum negative coefficients were located on the diagonal representing biological parent–child couples. (**b**) Spearman’s coefficients between parents (rows) and children (columns), as calculated for the positive feature vectors (i.e., positive CBI sub-model). Maximum correlation coefficients were located on the diagonal representing biological parent–child couples.

In a subsequent step, for both representations (also referred to as positive and negative CBI sub-models), biological parent–child couples were assigned to the diagonals of distance matrices that compared parental profiles with each of the children’s profiles. Following the identification rates, within these matrices, biological parent–child couples were characterized by the maximal Euclidean distances and the minimal negative correlation coefficients for the negative CBI sub-model (Fig. 8a, c). Similarly, biological couples sustained minimal Euclidean distances and maximal correlation coefficients for the positive features of the CBI sub-model (Fig. 8b, d). These results were also evident when applying the CBI model to the backward speech blocks as part of a sensitivity check (Supplementary Fig. 1).

**Figure 9:**
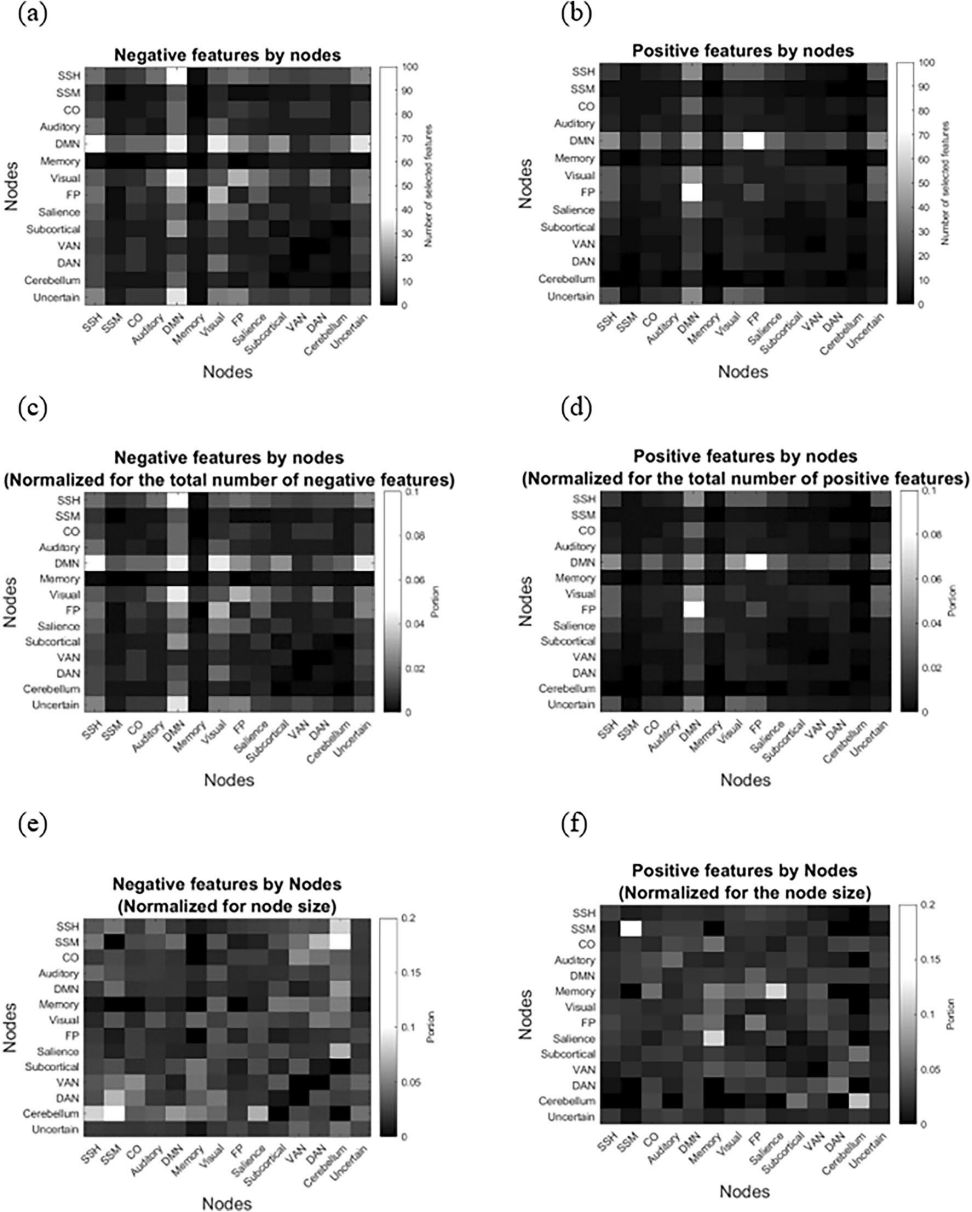
The contribution of various networks to the biological parent–child identification process. (**a**) The DMN-SSH (37 features), DMN-DMN (34 features), DMN-visual (34 features), and DMN-uncertain (33 features) contributed the majority of negative features to the negative model. (**b**) The DMN-FP node contributed most of the positive features to the positive model (72 features). (**c**) After normalization, the DMN-SSH (0.046), DMN-DMN (0.043), DMN-visual (0.043), and the DMN-uncertain (0.041) contributed the largest portions of features to the negative identification sub-model and (**d**) the DMN-FP contributed the largest portion of positive features (0.079). (**e**) The cerebellum-SSM node contributed the largest relative portion of features to the negative model, with a portion of 0.1. (**f**) The SSM-SSM (0.133), salience-memory (0.111), and cerebellum-cerebellum (0.1) nodes contributed the largest relative portion in the positive features model.

**Figure 10:**
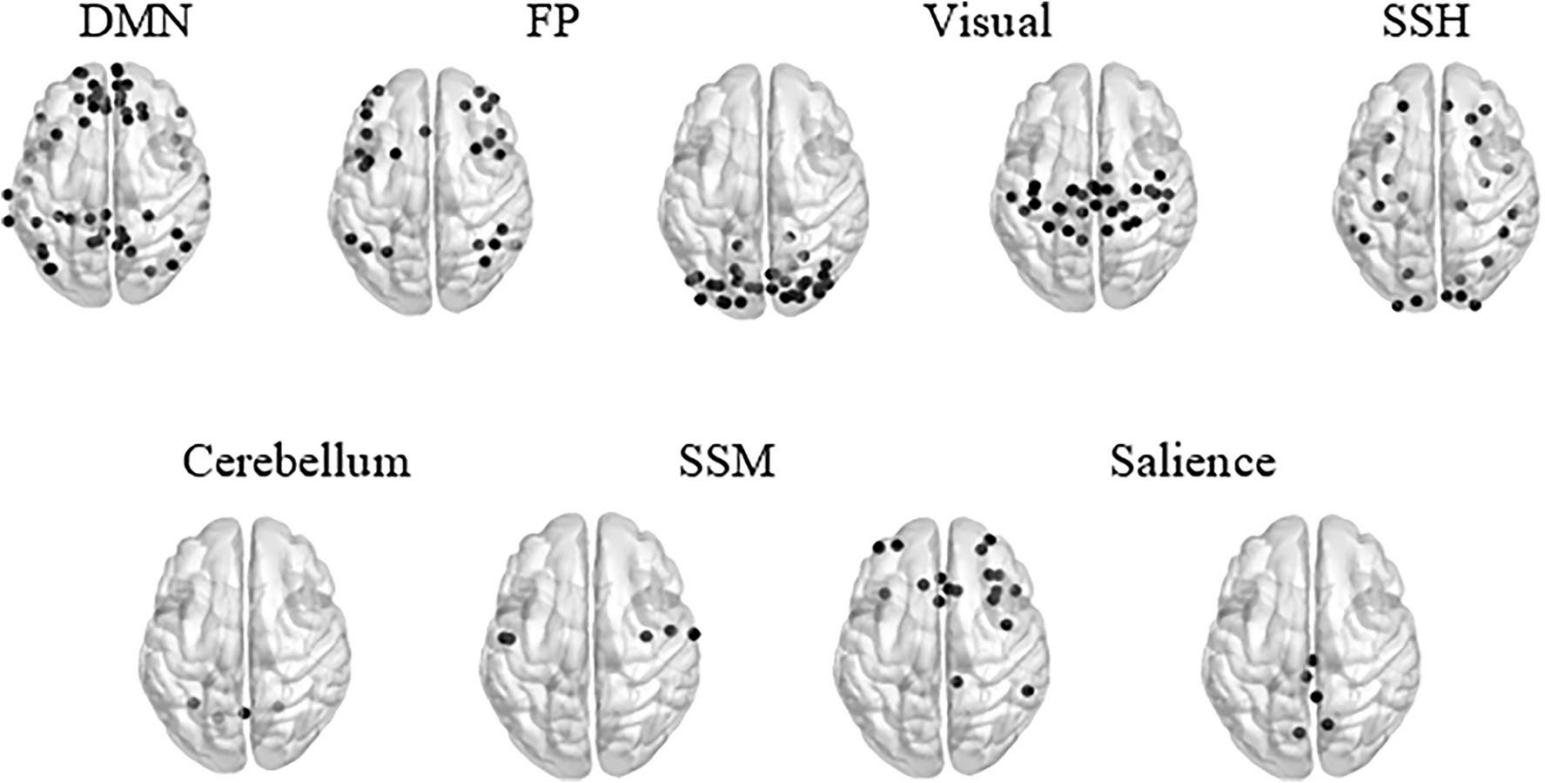
BrainNet Viewer^54^ illustration of the brain networks that enabled identifying biological parent–child couples. Top: the DMN-DMN, DMN-visual, DMN-SSH, and DMN-uncertain nodes contributed the highest portions of features to the negative sub-model, while the DMN-FP node contributed the highest portion to the positive sub-model. Bottom: illustration of the brain networks participating in the most correlative/anti-correlative parent–child neural correlates (fingerprints). The cerebellum-SSM node was the most anti-correlative node in the negative sub-model. The SSM-SSM, cerebellum-cerebellum, and salience-memory nodes introduced the most correlative patterns for the positive CBI sub-model.

In addition to the above, the results indicated that the DMN-somatosensory hand (SSH) (37 features), DMN-DMN (34 features), DMN-visual (34 features), and DMN-uncertain (33 features) contributed the majority of negative features to the negative CBI sub-model. Contrarily, the DMN-Fronto-Parietal (FP) node contributed the highest number of features (72 features) to the positive sub-model (Fig. 9a, b). To further investigate the contribution of each node (i.e., network-to-network connection) to the identification process, we normalized the number of negative/positive features within each node for the total number of negative/positive features included in the corresponding CBI sub-model. The DMN-SSH (0.046), DMN-DMN (0.043), DMN-visual (0.043), and the DMN-uncertain (0.041) contributed the largest portions of features to the negative identification sub-model. The DMN-FP contributed the largest portion (0.079) of positive features (Figs. 9c, d and 10) to the positive CBI sub-model. Finally, we normalized the number of within-node selected features by node size, to identify the nodes that were most correlated or anticorrelated for biological parent–child couples. Accordingly, the cerebellum-somatosensory mouth (SSM) node contributed the largest portion of features to the negative sub-model (0.1). The SSM-SSM (0.133), salience-memory (0.111), and the cerebellum-cerebellum (0.1) contributed the largest portions of features to the positive sub-model (Figs. 9e–f and 10). Overall, biological parent–child couples shared FC patterns that enabled their identification from a group of parents and children.

When conducting sensitivity analysis using the backward-speech blocks, the results indicated the identification of biological parent–child couples from a group of parents and children (see Supplemental data 1 for the full results).

### Diffusion maps demonstrate the singularity of biological parent–child couples

**Figure 11:**
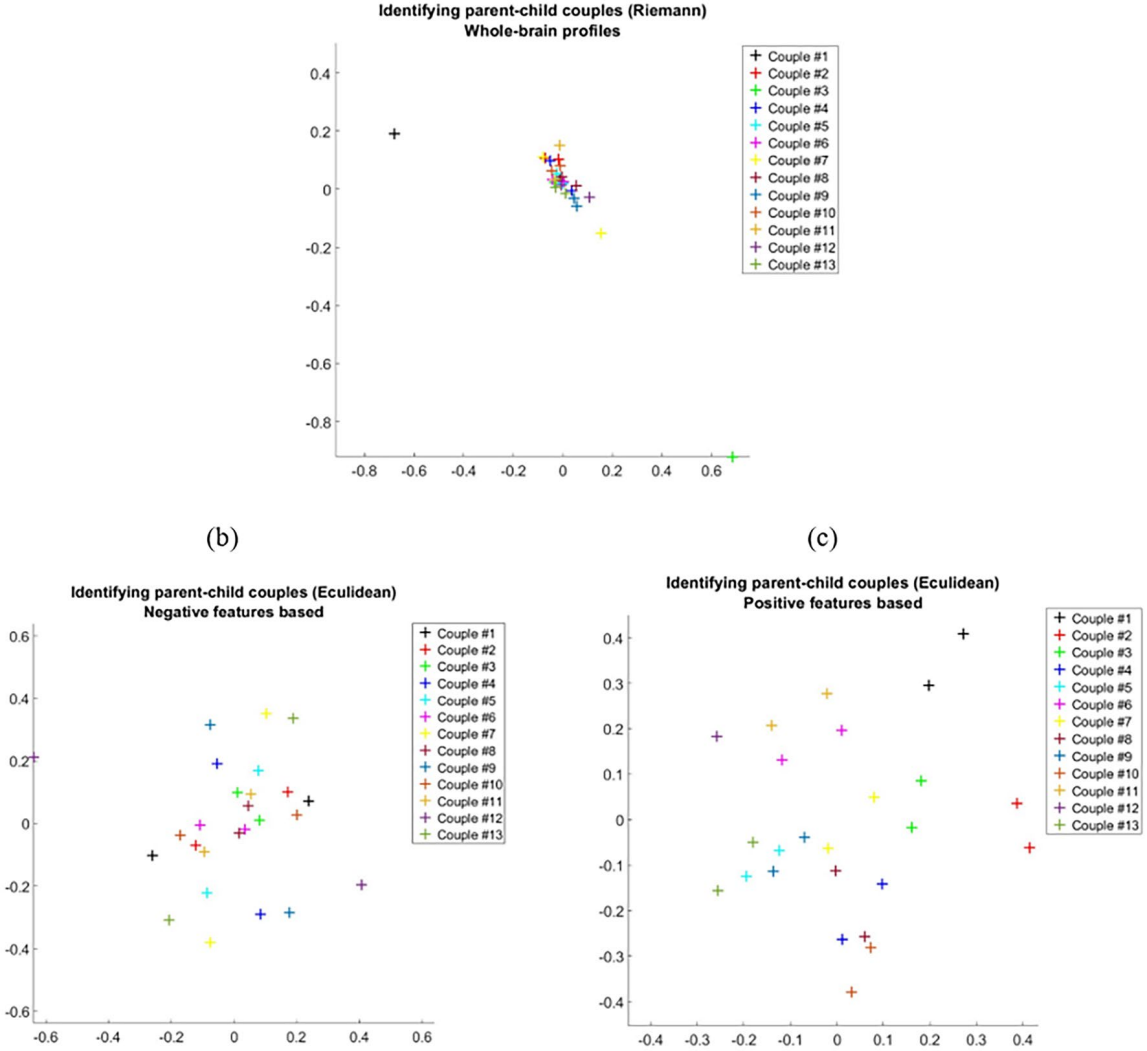
Identifying biological parent–child couples using diffusion maps**. (**a**) Whole-brain functional connectivity profiles obtained from parents and biological children were mapped to low-dimensional representation [i.e., diffusion maps (DM)], while preserving the Riemannian geometry of the manifold they lie on. Highly dense mapping in which biological parent–child couples (each couple is shown in the same color) were mapped with inconsistent proximity to each other reflected the results obtained for the fingerprinting model. (**b**) Euclidean DM obtained from the negative feature-based profiles demonstrated the mapping of parents and their biological children to distant coordinates, reflecting their distinct neural correlates. (**c**) For the Euclidean positive feature-based DM, biological parents and children of a dyad were consistently mapped close to each other, and more distant from other parents and children, reflecting the distinct parent–child brain similarities. The maps confirm connectome-based identification. **Author’s note: This figure must be colored.

We sought to investigate the distinct neural correlates associated with biological parent–child relations and the predictive capabilities of the fingerprinting and the CBI models. To this end, DMs were constructed for the whole-brain FC profiles, and for the positive and negative features-based representations used for the two CBI sub-models, respectively. Within those low-dimensional maps, 13 biological parent–child couples were represented by distinct colors. The DM constructed for the whole-brain profiles reflected the low prediction rates obtained for the fingerprinting model. This was evident in a dense mapping in which parent–child couples were not distinguished from other couples to indicate the singularity of their specific interaction (Fig. 11a). However, for the positive features-based profiles, the DM confirmed the results obtained for the positive CBI sub-model for identifying couples. More specifically, as hypothesized, biological parents and children of a couple were consistently mapped close to each other, and more distant from other parents and children. This indicates distinct FC similarities that form a neural fingerprint of a biological parent–child couple (Fig. 11b). In addition, when applied to the negative features-based profiles, the DM confirmed the results that were obtained for the negative CBI sub-model. Specifically, parents and their biological children were mapped to distant coordinates that reflected distinct neural differences associated with their interaction (Fig. 11c).

Finally, we conducted an unpaired Mann–Whitney test demonstrating the significant differences between biological couples and unrelated couples and indicated a significant difference with p-values of 2.072E−06 and 5.071E−6, respectively, confirming our hypotheses. The mean low-dimensional Euclidean distances between the DMs calculated for biological parent–child couples vs. unrelated couples were 0.132 (${\text{SE}} = 0.009$) versus 0.375 (${\text{SE}} = 0.014$), for the positive feature-based DM. The corresponding distances were 0.464 (${\text{SE }} = { }0.083$) versus 0.338 (${\text{SE}} = 0.014$), for the negative feature-based DM (i.e., biological couples were distinctively more distant from each other than were unrelated couples). The Mann–Whitney test did not reject the null hypothesis. While analyzing the control blocks as part of the sensitivity analysis, both tests rejected the null hypothesis, indicating significant differences between groups for both the positive and negative CBI sub-models. (Supplementary Fig. 3).

## Discussion

This study provides fMRI-based evidence for the existence of neural correlates for the relations within biological parent–child dyads. More specifically and in line with our hypotheses, intra-brain connectivity profiles recorded during story listening enabled accurate identification of biological parent–child couples. High identification rates were obtained for both positive and negative feature-based CBI sub-models according to their FC values. These results are consistent with studies that demonstrated increased brain-to-brain synchronization among individuals with similar characteristics^16,57,58^. Moreover, our findings are in line with studies that reported brain activation similarities while listening to stories, among socially connected people who shared similar perceptions and social behavior^25,26,59^.

Interestingly, for the positive feature-based sub-model, the highest number of parent–child FC similarities was within the DMN-FP node. Both these networks consisted of regions that were previously associated with social connectedness and narrative interpretation^26,32,57^, as well as regions related to the ‘gestalt cortex’. This cortex has been shown to be involved in generating immediate inferences for subjective perspectives^60^. Additionally, these inferences have been associated with attentional allocation regions, such as the superior and inferior parietal cortices, and regions associated with dynamic integration of incoming data (posterior cingulate cortex, precuneus, ventromedial prefrontal cortex, dorsomedial prefrontal cortex, bilateral temporoparietal cortex)^61^. Thus, brain similarities in FC between these two networks may reflect the shared perception that was evident in the interpretation of stories in the examined population.

We found that the SSM-SSM, salience-memory, and cerebellum-cerebellum nodes were the most correlative nodes that participated in the positive CBI sub-model (or neural fingerprint). These findings suggest similarity in the means that biological parents and children utilize cognitive and sensory brain networks that support cognitive monitoring, processing, and coordination of these skills (salience) while listening to stories. The cerebellum is known for balancing cognitive tasks^14,62^ and was previously related to learning (especially in the linguistic domain^63,64^) and hence, we postulate that the parental cerebellum plays a role in tuning a child’s language processing during listening comprehension. Additionally, we suggest that similar engagement of the salience-memory retrieval node indicates that parent–child couples similarly retrieve information from memory to support comprehension of stories. Finally, FC similarities within the SSM network indicate similarity in language perception that supports comprehension of stories by biological parent–child couples^65^.

The highest number of anti-correlative features that were included in the negative feature-based CBI sub-model were within the DMN-SSH, DMN-DMN, DMN-visual, and DMN-uncertain nodes. This suggests that the biological parent–child interaction patterns observed also involved distinct anti-correlative connectivity patterns. These patterns mainly involved the DMN, but also sensory (SSH, visual) and limbic-related networks (here labeled as the uncertain network^53^). Previous work^15^ implied positive associations of similarities in connectivity patterns obtained from parents and children, with children’s age. The negative functional connection of DMN during story listening was suggested as an indicator of maturation and attention to the story^66^. We postulate that these anti-correlative FC patterns reflect a distinct brain mechanism that is associated with the exposure of biological parent-children couples to the same stories. These patterns may reflect a parent’s brain tuning toward a child’s execution of complex cognitive and social processes.

An alternative explanation is based on the literature previously discussing the relations between parental cognitive control and emotional regulation^9^. For example, greater parental cognitive control (i.e. activation of the prefrontal cortex in the parent) might be related to reduced activation of limbic-related regions in the child (resembling better emotion regulation in the child). In the context of the current study, the anti-correlation results focused on negative FC between cognitive networks (DMN-DMN), cognitive-sensory networks (DMN-SSH, DMN-vis), or cognitive-limbic networks (DMN-uncertain, reflecting the limbic system). Similarly, we suggest that greater parental cognitive control might be related to reduced effort demands from the child while processing a narrative. Therefore, we find anti-correlation with cognitive, limbic and sensory networks in the child. Additional studies should examine the relations between these parent–child anti-correlations and cognitive control/emotional regulation of parent–child dyads. The engagement of these networks, however, was specific to the story listening condition (and not for the control, backward speech condition). A possible interpretation is that the story listening condition engages cognitive and language processing as well as visual processing engagement, whereas backward speech does not.

What caused the CBI model to outperform the fingerprint model? The fingerprinting and the proposed CBI models demonstrate how similarities in functional connectivity (as identified within-person or across individuals) act as predictive neural signatures. Theoretically, for both models, these neural signatures (‘fingerprints’) can be identified from whole-brain functional connectivity profiles or from specific network-to-network nodes. However, with few studies utilizing fMRI data to investigate neural similarities between parents and children, we did not limit the current analyses to specific nodes. Thus, both models were fed with two whole-brain parent-only and child-only sets. For the fingerprinting model, whole-brain profiles were correlated across groups (sets) to identify/predict biological parent–child couples. For the CBI model, the values of each ROI-to-ROI connection were correlated across groups to extract features. We suggest that these features were assumed to embed hidden information about the relationship between both functional sets. Then, they were used to represent each individual within the two sets. Importantly, to hold meaningful analysis, we distinguished between positive and negative features before correlating or calculating the Euclidean distance between parental and children’s representations.

We suggest that these features were assumed to embed hidden information about the relationship between both functional sets. Then, they were used to represent each individual within the two sets. Importantly, to hold meaningful analysis, we distinguished between positive and negative features before correlating or calculating the Euclidean distance between parental and children’s representations.

Overall, the two CBI sub-models confirmed that biological parent–child couples share FC similarities and differences that may serve as an fMRI-based neural fingerprint. Our findings concur the importance of parent–child relations in developing children’s cognitive and social skills^1–8^, and imply the distinct neural synchronization that characterizes biological parent–child couples. Interestingly, a similar pattern was found in the control backward-speech condition, which may suggest that the neurobiological correlates for directing attention to an auditory stimulus (in this case- backward speech) are very similar in children and their biological parents. The extent to which environmental and genetic factors contribute to connectome similarity is still unknown and requires further research.

The DM obtained in the current study successfully demonstrated the existence of a neural fingerprint for distinct biological parent–child couples, thus corroborating studies that adapted the DM framework to investigate brain-behavior relations^28,29^. The graphical representations revealed in the current study enabled identifying parent–child couples and supported the results of the fingerprinting^20^ and the CBI models. Specifically, when applied to the whole-brain connectivity profiles that correspond to the fingerprinting model, dense mapping did not indicate any distinct neural fingerprints for biological parent–child couples. However, the DM obtained for the positive and negative feature-based CBI sub-models demonstrated the existence of neural fingerprints that represent particular biological parent–child couples. More specifically, for the positive feature-based DM, biological parent–child couples maintained minimal Euclidean distances and greater distances from other couples. This result is supported by the mean low-dimensional distances associated with biological parent–child couples vs. unrelated couples and the corresponding standard errors. This also indicated that biological parent–child couples were characterized by distinct minimal proximity between their FC patterns within specific brain regions. Additionally, for the negative feature-based DM, couples were mapped to distinct coordinates. This indicates that biological parent–child couples maintained a maximal Euclidean distance between FC patterns within other brain regions. This less intuitive representation provides additional evidence for the distinct brain correlates that are associated with biological parent–child relations and that should be considered a transformation of the parent–child neural fingerprint. Importantly, this result was also supported by the mean values calculated for the low-dimensional distances associated with biological parent–child couples vs. unrelated couples and their corresponding standard errors.

Finally, based on the current study’s dataset and the literature, as well as the sensitivity analysis, we argue that while preserving the data structure without making any assumptions about its nature, the DM and the CBI should be considered frameworks for measuring brain-behavior relations. The CBI model is a purely brain-driven method that does not require any a-priori assumptions about the data, including the investigated sample size or the scanning duration. Thus, whether combined with DM or not, the model can be applied to other populations and modalities. Indeed, due to the inherent nature of the DM framework, which enables classification^30^, the two frameworks afford expanding the study of brain-behavior relations to other behavioral, functional, and clinical measurements. For instance, we suggest combining the CBI and DM frameworks to measure within-group brain profile proximity before and after interventions and to assess pathological progress^28^. Our findings can be further generalized to a non-specific task condition (e.g., resting state). Nonetheless, they open opportunities to link similarities and differences in brain connectivity to additional behavioral, psychological, and medical phenomena in biologically-related individuals and other populations.

## Study limitations

This work has several limitations that need to be taken into account. First, the investigated data included only 13 biological parent–child couples. The small sample size was accounted for by applying appropriate statistical tools, including permutation testing.

In addition, the parental cohort investigated in the current study included 11 females and 2 males with above-average socioeconomic status, which may have affected our results. Notably, the present study explored parent–child neural correlates from a network view. Thus, the specific brain regions that are involved in the distinct parent–child neural fingerprint remain unknown.

Another point is that before preprocessing the data, 30-s-long story blocks were aggregated. Performing the concatenation before the preprocessing could have affected the movement correction phase in the merge phase. However, due to the consistency across all the participants, we argue that this procedure did not distort the results. Moreover, the analysis described in this paper contained 150 s (i.e. 150 volumes, TR = 1 s). Although more data is always beneficial for functional connectivity analysis, we and others have previously demonstrated stable, functional connectivity matrices using a similar task in the same length^6,67,68^.

Finally, this study examined biological parent–child brain coupling while parents and children listened to the same stories separately. Therefore, the extracted neural fingerprints described brain coupling that corresponded to a story-listening task, as measured in a ‘pseudo-synchronized’ manner.

## Future research

Future studies should expand the current examination, first to other task conditions (e.g., the resting state) and then to an fMRI hyper scan that involves parents and children interacting with each other during the scan (e.g., while listening to stories, watching movies, or during a conversation). This may enable the identification of robust parent–child neural fingerprints that resemble parent–child relations. As the central adults in children’s lives, parents tune their children’s cognitive and social skills^1^. Future studies should investigate how the quality of parent–child interactions shapes neural fingerprints, also compared to genetic factors.

In addition to the above, the algorithms used in the current study can be applied to investigate whole-brain or network-oriented profiles that are acquired using various modalities (e.g., electroencephalogram, fMRI, functional near-infrared spectroscopy) to refine brain-behavior relations. For example, as the DM can include several signal sources that are associated with parent–child interactions (motion, voice, etc.), these sources can be added in future studies to refine the characteristics of parent–child neural synchronization. Moreover, the data acquisition in this study followed a block-design configuration. Drawing from previous studies that have shown no significant differences in the interpretation of functional connectivity data between the concatenation of blocks and continuous acquisition^48–50^ the current study adopted block aggregation. However, it would be interesting to examine the differences in biological dyad identification in data acquired over minutes vs block design.

Finally, the proposed frameworks enable linking brain connectivity patterns to specific behavioral, psychological, and pathological aspects, also among other populations.

## Conclusions

Biological parent–child couples demonstrated distinct functional correlates while listening to stories. These correlates were evident in both cognitive and sensory networks. We argue that brain synchronization while listening to stories characterizes biological parent–child relationships. The CBI and DM frameworks proposed here add to the existing knowledge about brain mechanisms that underly biological parent–child relations and highlight the variability between parent–child couples. To our knowledge, this is the first study to reveal the neural fingerprint that represents distinct biological parent–child couples. Defining “typical” neural correlates for parent–child relations will enable preventing impaired connection due to parental depression or child-related disorders (language or social challenges)**.** We argue that once “typical” biological parent–child connections are defined, this can set up the landmark for additional studies utilizing the same analytic approach to identify biological parent-children whose connectome similarity does not show proximity as expected. These dyads could then be followed up and provided with appropriate interventions to provide tools to improve parent–child interaction.

### Supplementary Information

Supplementary Information.

## Data Availability

Data and codes are available upon request from the corresponding author (Horowitz-Kraus).
