# Supplementary material for: Parent–child couples display shared neural fingerprints while listening to stories
Source: Sci Rep. 2024 Feb 4;14:2883. doi: 10.1038/s41598-024-53518-x (PMC10838923; doi:10.1038/s41598-024-53518-x)
Supplement: Supplementary file 1 — Supplementary Information. [file 41598_2024_53518_MOESM1_ESM.docx]

**Supplementary material**

**Supplementary data 1:**

**The control condition analysis (backward speech)**

**Methods**

### Additional sensitivity analysis (control blocks)

An additional sensitivity analysis was conducted by applying the proposed tools to the concatenated five 30-second-long backward speech control blocks preprocessed before. It was hypothesized that if biological parent-child couples displayed shared neural fingerprints while listening to stories, they would also display it during a backward speech stimulation. However, it was also hypothesized that the fingerprinting features were somewhat different since they were also determined by the different scanning conditions.

**Results**

For the fingerprinting model, the whole-brain connectivity profiles of all 26 parents and children were assigned to ‘target’ and ‘database’ sets ^12^. Each set consisted of 13 parent-only or child-only FC profiles. Identification rates were measured for the target-database of children-parents and its reverse form, i.e., parents-children. Similarly to the task blocks, the identification rates were 15.4% (2/13 dyads) and 7.7% (1/13 dyads), respectively, thus obviating the need for additional significance tests. These results indicated that for the control blocks the fingerprinting method failed to identify biological parent-child as well.

When applying the suggested CBI model to the control blocks, the identification rates for both the Euclidean metric and Spearman’s correlations were 100% (13/13 couples) for the positive and negative feature-based representations (see supplementary Fig. 1). These results were then verified through the first permutation testing in which each group was shuffled. After shuffling, the mean identification rates for the Euclidean distance-based method dropped to 7.734% (~1/13 dyads) and 7.963% (~1/13 dyads) for the positive and negative features, respectively. The mean identification rates for the Spearman-based method dropped to 7.734% (~1/13 dyads) and 7.66% (~1/13 dyads) for the positive and negative features, respectively, indicating a zero non-parametric
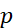
 value.

For the second permutation test, the two sets of parents and children were shuffled on each iteration. Then, Spearman’s correlation with a significance threshold of 0.05 was performed across the two random sets to iteratively obtain positive and negative features. These features were then used to represent each participant (parent/child) vector-wise within the original parent-only and child-only sets based on their own FC data, and identification rates were measured per the new representations.

Per the control blocks, for the Euclidean distances, the maximum identification rate measured was 46.154% (6/13 couples) occurring in 0.0004% (2/5,000) of the permutations for the positive features, and 23.077% (3/13 couples) occurring in 0.0128% (64/5,000) of the iterations for the negative features. Mean identification rates were 1.033 and 0.756 accurate couples, respectively. For the Spearman’s coefficients, the maximum identification rate measured was 53.846% (7/13 couples) occurring in 0.0002% (1/5,000) of the permutations for the positive features, and 38.462% (5/13 couples) occurring in 0.0004% (2/5,000) of the iterations for the negative features. Mean identification rates were 0.882 and 1.157 accurate couples, respectively. These results also indicated low identification rates compared to those obtained when identifying couples upon correlation across the unshuffled sets, thus strengthening the results.

When conducting sensitivity analysis using the backward-speech blocks, the results indicated that the DMN-visual (45 features), DMN- FP (36 features), DMN-uncertain (30 features), visual-uncertain (30 features), DMN-SSH (somatosensory hand), and the visual-FP nodes (27 features each), contributed the majority of negative features to the negative CBI sub-model. However, the DMN-DMN and the DMN-SSH nodes contributed the highest number of features (72 and 52 features, respectively) to the positive sub-model (Supplementary Fig. 2a-b). When normalizing the number of negative/positive features within each node for the total number of negative/positive features included in the corresponding CBI sub-model, the DMN-visual (0.063), DMN- FP (0.051), DMN-uncertain (0.042), visual-uncertain (0.042), DMN-SSH (somatosensory hand) , and the visual-FP nodes (0.038), contributed the largest portions of features to the negative identification sub-model. The DMN-DMN (0.07) and the DMN-SSH (0.051) nodes contributed the largest portions of positive features (Supplementary Fig. 2c-d) to the positive CBI sub-model. Finally, we normalized the number of within-node selected features by node size to identify the nodes that were most correlated or anticorrelated for biological parent-child couples. Accordingly, the VAN-memory node contributed the largest portion of features to the negative sub-model (0.089). The SSM-memory (0.16), and the SSM-SSM (0.133), contributed the largest portions of features to the positive sub-model (Supplementary Fig.2e-f). Overall, analyzing the control blocks enabled biological parent-child identification couples from a group of parents and children.

The control DMs also supported our findings: The DM constructed for the whole-brain FC profiles obtained while parents and biological children were listening to backward speech (i.e., the control blocks), reflected the low prediction rates obtained for the fingerprinting model. This was evident in a dense mapping in which parent-child couples were not distinguished from other couples to indicate the singularity of their specific interaction (Sup. Fig. 3a). However, for the positive features-based profiles, the DM confirmed the results obtained for the positive CBI sub-model for identifying couples. More specifically, as hypothesized, biological parents and children of a dyad were consistently mapped close to each other and more distant from other parents and children. This indicates distinct FC similarities that form a task-independent neural fingerprint of a biological parent-child couple (Sup. Fig. 3b). In addition when applied to the negative features-based profiles, the DM confirmed the results that were obtained for the negative CBI sub-model. Specifically, parents and their biological children were mapped to distant coordinates that reflected distinct neural differences associated with their interaction (Sup. Fig. 3c). Finally, confirming our hypotheses, the mean low-dimensional Euclidean distances between the DMs calculated for biological parent-child couples vs. unrelated couples were 0.151 ($SE=0.018$) vs. 0.374 ($SE=0.015$), for the positive feature-based DM (i.e., biological couples were distinctively closer to each other than were unrelated couples). The corresponding distances were 0.504 ($SE = 0.062$) vs. 0.348($SE=0.012$), for the negative feature-based DM (i.e., biological couples were distinctively more distant from each other than were unrelated couples).

When conducting sensitivity analysis using the backward-speech blocks, the results indicated the DMN-visual (45 features), DMN-FP (36 features), DMN-uncertain (30 features), visual-uncertain (30 features), DMN-SSH (somatosensory hand), and the visual-FP nodes (27 features each), contributed the majority of negative features to the negative CBI sub-model. However, the DMN-DMN and the DMN-SSH nodes contributed the highest number of features (72 and 52 features, respectively) to the positive sub-model (Supplementary Fig. 2a-b). When normalizing the number of negative/positive features within each node for the total number of negative/positive features included in the corresponding CBI sub-model, the DMN-visual (0.063), DMN- FP (0.051), DMN-uncertain (0.042), visual-uncertain (0.042), DMN-SSH (somatosensory hand), and the visual-FP nodes (0.038), contributed the largest portions of features to the negative identification sub-model. The DMN-DMN (0.07) and the DMN-SSH (0.051) nodes contributed the largest portions of positive features (Supplementary Fig. 2c-d) to the positive CBI sub-model. Finally, we normalized the number of within-node selected features by node size to identify the most correlated or anticorrelated nodes for biological parent-child couples. Accordingly, the VAN-memory node contributed the largest portion of features to the negative sub-model (0.089). The SSM-memory (0.16), and the SSM-SSM (0.133), contributed the largest portions of features to the positive sub-model (Supplementary Fig.2e-f). Overall, analyzing the control blocks enabled biological parent-child identification couples from a group of parents and children.

**Supplementary Figures**


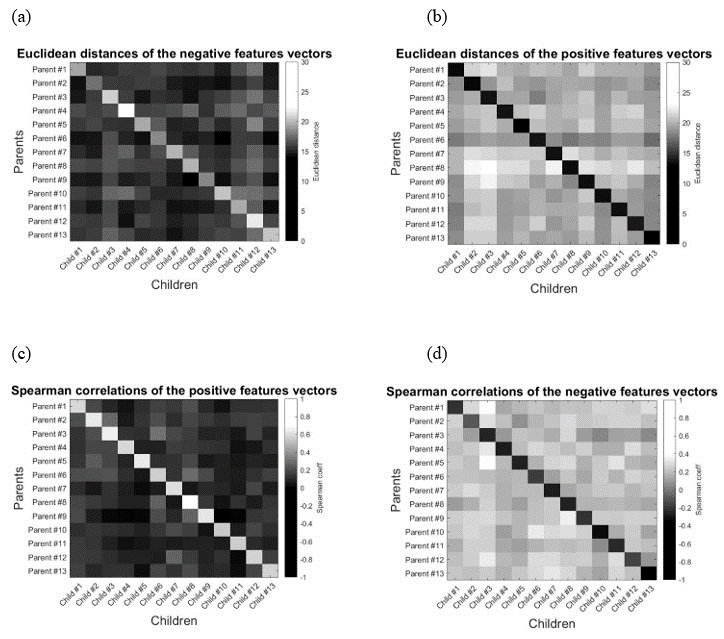


**Supplementary Fig. 1.** **The Connectome-based Identification (CBI) results per control blocks.** (a) The Euclidean distance between parents (rows) and children (columns), as calculated for the negative feature vectors (i.e., negative CBI sub-model). The maximum distances were located on the diagonal, representing biological parent-child couples. (b) The Euclidean distance between the parents (rows) and children (columns), as calculated for the positive feature vectors (i.e., positive CBI sub-model). Minimum distances were located on the diagonal, representing biological parent-child couples. (c) Spearman’s correlation coefficients between parents (rows) and children (columns), as calculated for the negative feature vectors (i.e., negative CBI sub-model). Minimum negative coefficients were located on the diagonal representing biological parent-child couples. (b) Spearman’s coefficients between parents (rows) and children (columns), as calculated for the positive feature vectors (i.e., positive CBI sub-model). Maximum correlation coefficients were located on the diagonal representing biological parent-child couples.


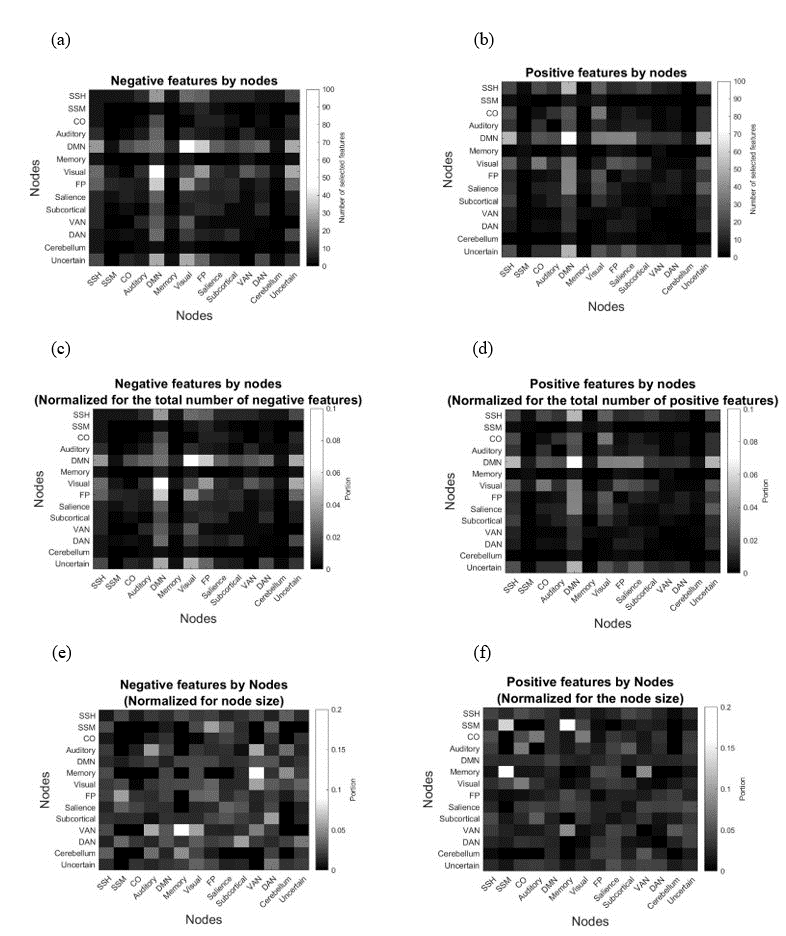


**Supplementary Fig. 2.** **Sensitivity analysis:** **The contribution of various networks to the biological parent-child identification process while listening to backward speech.** (a) The DMN-visual (45 features), DMN- FP (36 features), DMN-uncertain (30 features), visual-uncertain (30 features), DMN-SSH (somatosensory hand), and the visual-FP nodes (27 features each), contributed the majority of negative features to the negative CBI sub-model. (b) The DMN-DMN and the DMN-SSH nodes contributed the highest number of features (72 and 52 features, respectively) to the positive sub-model. (c) After normalization, the DMN-visual (0.063), DMN-FP (0.051), DMN-uncertain (0.042), visual-uncertain (0.042), DMN-SSH (somatosensory hand), and the visual-FP nodes (0.038), contributed the largest portions of features to the negative identification sub-model (d) The DMN-DMN (0.07) and the DMN-SSH (0.051) nodes contributed the largest portions of positive features to the positive CBI sub-model. (e) The VAN-memory node contributed the largest portion of features to the negative sub-model (0.089). (f) The SSM-memory (0.16), and the SSM-SSM (0.133), contributed the largest portions of features to the positive sub-model.


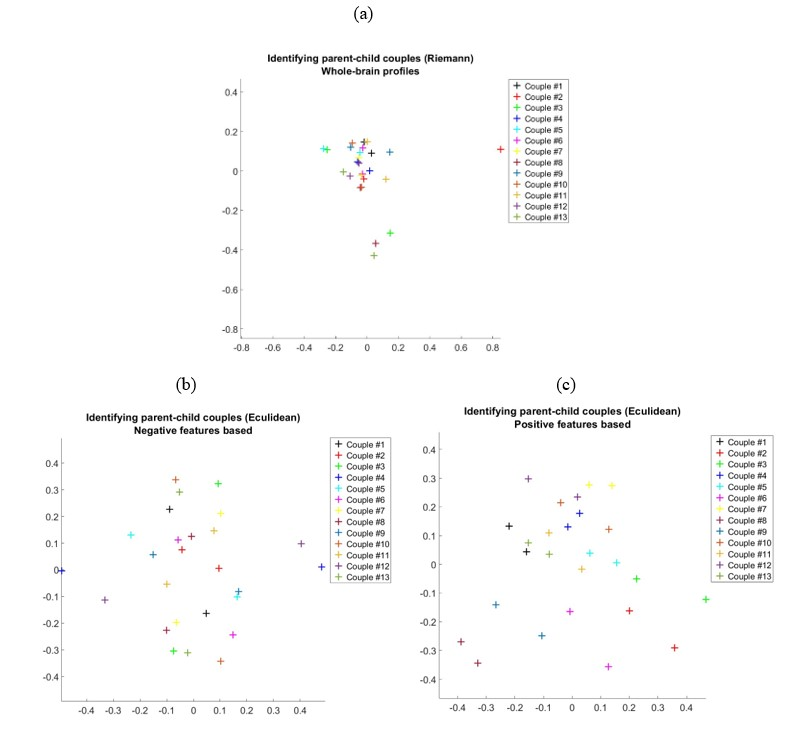


****Supplementary Fig. 3.** **Sensitivity check:** **Identifying biological parent-child couples using diffusion maps**.** (a) Whole-brain functional connectivity profiles obtained from parents and biological children were mapped to low-dimensional representation [i.e., diffusion maps (DM)], while preserving the Riemannian geometry of the manifold they lie on. Highly dense mapping in which biological parent-child couples (each couple is shown in the same color) were mapped with inconsistent proximity to each other reflected the results obtained for the fingerprinting model. (b) Euclidean DM obtained from the negative feature-based profiles demonstrated the mapping of parents and their biological children to distant coordinates, reflecting their distinct neural correlates. (c) For the Euclidean positive feature-based DM, biological parents and children of a dyad were consistently mapped close to each other, and more distant from other parents and children, reflecting the distinct parent-child brain similarities. The maps confirm connectome-based identification.

**Author’s note: This figure must be colored

| **Class** | **Network** | **Anatomical regions** | **MNI coordinates** | | |
| --- | --- | --- | --- | --- | --- |
|  |  |  | **x** | **y** | **z** |
| Sensory | Sensory/somatomotor Hand | 'Precuneus' | -7 | -52 | 61 |
|  | Sensory/somatomotor Hand | 'Cingulate Gyrus' | -14 | -18 | 40 |
|  | Sensory/somatomotor Hand | 'Paracentral Lobule' | 0 | -15 | 47 |
|  | Sensory/somatomotor Hand | 'Cingulate Gyrus' | 10 | -2 | 45 |
|  | Sensory/somatomotor Hand | 'Medial Frontal Gyrus' | -7 | -21 | 65 |
|  | Sensory/somatomotor Hand | 'Postcentral Gyrus' | -7 | -33 | 72 |
|  | Sensory/somatomotor Hand | 'Postcentral Gyrus' | 13 | -33 | 75 |
|  | Sensory/somatomotor Hand | 'Postcentral Gyrus' | -54 | -23 | 43 |
|  | Sensory/somatomotor Hand | 'Precentral Gyrus' | 29 | -17 | 71 |
|  | Sensory/somatomotor Hand | 'Postcentral Gyrus' | 10 | -46 | 73 |
|  | Sensory/somatomotor Hand | 'Postcentral Gyrus' | -23 | -30 | 72 |
|  | Sensory/somatomotor Hand | 'Postcentral Gyrus' | -40 | -19 | 54 |
|  | Sensory/somatomotor Hand | 'Postcentral Gyrus' | 29 | -39 | 59 |
|  | Sensory/somatomotor Hand | 'Postcentral Gyrus' | 50 | -20 | 42 |
|  | Sensory/somatomotor Hand | 'undefined' | -38 | -27 | 69 |
|  | Sensory/somatomotor Hand | 'Precentral Gyrus' | 20 | -29 | 60 |
|  | Sensory/somatomotor Hand | 'Precentral Gyrus' | 44 | -8 | 57 |
|  | Sensory/somatomotor Hand | 'Postcentral Gyrus' | -29 | -43 | 61 |
|  | Sensory/somatomotor Hand | 'Superior Frontal Gyrus' | 10 | -17 | 74 |
|  | Sensory/somatomotor Hand | 'Postcentral Gyrus' | 22 | -42 | 69 |
|  | Sensory/somatomotor Hand | 'Inferior Parietal Lobule' | -45 | -32 | 47 |
|  | Sensory/somatomotor Hand | 'Postcentral Gyrus' | -21 | -31 | 61 |
|  | Sensory/somatomotor Hand | 'Precentral Gyrus' | -13 | -17 | 75 |
|  | Sensory/somatomotor Hand | 'Postcentral Gyrus' | 42 | -20 | 55 |
|  | Sensory/somatomotor Hand | 'undefined' | -38 | -15 | 69 |
|  | Sensory/somatomotor Hand | 'Postcentral Gyrus' | -16 | -46 | 73 |
|  | Sensory/somatomotor Hand | 'Medial Frontal Gyrus' | 2 | -28 | 60 |
|  | Sensory/somatomotor Hand | 'Medial Frontal Gyrus' | 3 | -17 | 58 |
|  | Sensory/somatomotor Hand | 'Precentral Gyrus' | 38 | -17 | 45 |
|  | Sensory/somatomotor Hand | 'Postcentral Gyrus' | 47 | -30 | 49 |
|  | | | | | |
| Sensory | Sensory/somatomotor Mouth | 'Precentral Gyrus' | -49 | -11 | 35 |
|  | Sensory/somatomotor Mouth | 'Insula' | 36 | -9 | 14 |
|  | Sensory/somatomotor Mouth | 'Precentral Gyrus' | 51 | -6 | 32 |
|  | Sensory/somatomotor Mouth | 'Precentral Gyrus' | -53 | -10 | 24 |
|  | Sensory/somatomotor Mouth | 'Precentral Gyrus' | 66 | -8 | 25 |
|  | | | | | |
| Cognitive | Cingulo-opercular Task Control | 'Medial Frontal Gyrus' | -3 | 2 | 53 |
|  | Cingulo-opercular Task Control | 'Inferior Parietal Lobule' | 54 | -28 | 34 |
|  | Cingulo-opercular Task Control | 'Middle Frontal Gyrus' | 19 | -8 | 64 |
|  | Cingulo-opercular Task Control | 'Superior Frontal Gyrus' | -16 | -5 | 71 |
|  | Cingulo-opercular Task Control | 'Cingulate Gyrus' | -10 | -2 | 42 |
|  | Cingulo-opercular Task Control | 'Insula' | 37 | 1 | -4 |
|  | Cingulo-opercular Task Control | 'Superior Frontal Gyrus' | 13 | -1 | 70 |
|  | Cingulo-opercular Task Control | 'Medial Frontal Gyrus' | 7 | 8 | 51 |
|  | Cingulo-opercular Task Control | 'Precentral Gyrus' | -45 | 0 | 9 |
|  | Cingulo-opercular Task Control | 'Superior Temporal Gyrus' | 49 | 8 | -1 |
|  | Cingulo-opercular Task Control | 'Claustrum' | -34 | 3 | 4 |
|  | Cingulo-opercular Task Control | 'Superior Temporal Gyrus' | -51 | 8 | -2 |
|  | Cingulo-opercular Task Control | 'Cingulate Gyrus' | -5 | 18 | 34 |
|  | Cingulo-opercular Task Control | 'Insula' | 36 | 10 | 1 |
|  | | | | | |
| Sensory | Auditory | 'Insula' | 32 | -26 | 13 |
|  | Auditory | 'Superior Temporal Gyrus' | 65 | -33 | 20 |
|  | Auditory | 'Superior Temporal Gyrus' | 58 | -16 | 7 |
|  | Auditory | 'Insula' | -38 | -33 | 17 |
|  | Auditory | 'Superior Temporal Gyrus' | -60 | -25 | 14 |
|  | Auditory | 'Superior Temporal Gyrus' | -49 | -26 | 5 |
|  | Auditory | 'Insula' | 43 | -23 | 20 |
|  | Auditory | 'Inferior Parietal Lobule' | -50 | -34 | 26 |
|  | Auditory | 'Postcentral Gyrus' | -53 | -22 | 23 |
|  | Auditory | 'Precentral Gyrus' | -55 | -9 | 12 |
|  | Auditory | 'Precentral Gyrus' | 56 | -5 | 13 |
|  | Auditory | 'Postcentral Gyrus' | 59 | -17 | 29 |
|  | Auditory | 'Insula' | -30 | -27 | 12 |
|  | | | | | |
| Cognitive | Default mode | 'Middle Temporal Gyrus' | -41 | -75 | 26 |
|  | Default mode | 'undefined' | 6 | 67 | -4 |
|  | Default mode | 'Medial Frontal Gyrus' | 8 | 48 | -15 |
|  | Default mode | 'Parahippocampa Gyrus' | -13 | -40 | 1 |
|  | Default mode | 'Superior Frontal Gyrus' | -18 | 63 | -9 |
|  | Default mode | 'Middle Temporal Gyrus' | -46 | -61 | 21 |
|  | Default mode | 'Middle Temporal Gyrus' | 43 | -72 | 28 |
|  | Default mode | 'Superior Temporal Gyrus' | -44 | 12 | -34 |
|  | Default mode | 'Superior Temporal Gyrus' | 46 | 16 | -30 |
|  | Default mode | 'undefined' | -68 | -23 | -16 |
|  | Default mode | 'Angular Gyrus' | -44 | -65 | 35 |
|  | Default mode | 'undefined' | -39 | -75 | 44 |
|  | Default mode | 'Cingulate Gyrus' | -7 | -55 | 27 |
|  | Default mode | 'Precuneus' | 6 | -59 | 35 |
|  | Default mode | 'Posterior Cingulate' | -11 | -56 | 16 |
|  | Default mode | 'Posterior Cingulate' | -3 | -49 | 13 |
|  | Default mode | 'Cingulate Gyrus' | 8 | -48 | 31 |
|  | Default mode | 'Precuneus' | 15 | -63 | 26 |
|  | Default mode | 'Cingulate Gyrus' | -2 | -37 | 44 |
|  | Default mode | 'Posterior Cingulate' | 11 | -54 | 17 |
|  | Default mode | 'Angular Gyrus' | 52 | -59 | 36 |
|  | Default mode | 'Middle Frontal Gyrus' | 23 | 33 | 48 |
|  | Default mode | 'Superior Frontal Gyrus' | -10 | 39 | 52 |
|  | Default mode | 'Superior Frontal Gyrus' | -16 | 29 | 53 |
|  | Default mode | 'Middle Frontal Gyrus' | -35 | 20 | 51 |
|  | Default mode | 'Superior Frontal Gyrus' | 22 | 39 | 39 |
|  | Default mode | 'Superior Frontal Gyrus' | 13 | 55 | 38 |
|  | Default mode | 'Superior Frontal Gyrus' | -10 | 55 | 39 |
|  | Default mode | 'Superior Frontal Gyrus' | -20 | 45 | 39 |
|  | Default mode | 'Medial Frontal Gyrus' | 6 | 54 | 16 |
|  | Default mode | 'Medial Frontal Gyrus' | 6 | 64 | 22 |
|  | Default mode | 'Medial Frontal Gyrus' | -7 | 51 | -1 |
|  | Default mode | 'Medial Frontal Gyrus' | 9 | 54 | 3 |
|  | Default mode | 'Medial Frontal Gyrus' | -3 | 44 | -9 |
|  | Default mode | 'Medial Frontal Gyrus' | 8 | 42 | -5 |
|  | Default mode | 'Medial Frontal Gyrus' | -11 | 45 | 8 |
|  | Default mode | 'Medial Frontal Gyrus' | -2 | 38 | 36 |
|  | Default mode | 'Anterior Cingulate' | -3 | 42 | 16 |
|  | Default mode | 'Superior Frontal Gyrus' | -20 | 64 | 19 |
|  | Default mode | 'Medial Frontal Gyrus' | -8 | 48 | 23 |
|  | Default mode | 'Inferior Temporal Gyrus' | 65 | -12 | -19 |
|  | Default mode | 'Middle Temporal Gyrus' | -56 | -13 | -10 |
|  | Default mode | 'Middle Temporal Gyrus' | -58 | -30 | -4 |
|  | Default mode | 'Middle Temporal Gyrus' | 65 | -31 | -9 |
|  | Default mode | 'Middle Temporal Gyrus' | -68 | -41 | -5 |
|  | Default mode | 'Superior Frontal Gyrus' | 13 | 30 | 59 |
|  | Default mode | 'Anterior Cingulate' | 12 | 36 | 20 |
|  | Default mode | 'Middle Temporal Gyrus' | 52 | -2 | -16 |
|  | Default mode | 'Parahippocampa Gyrus' | -26 | -40 | -8 |
|  | Default mode | 'Parahippocampa Gyrus' | 27 | -37 | -13 |
|  | Default mode | 'Fusiform Gyrus' | -34 | -38 | -16 |
|  | Default mode | 'Uvula' | 28 | -77 | -32 |
|  | Default mode | 'Middle Temporal Gyrus' | 52 | 7 | -30 |
|  | Default mode | 'Middle Temporal Gyrus' | -53 | 3 | -27 |
|  | Default mode | 'Supramarginal Gyrus' | 47 | -50 | 29 |
|  | Default mode | 'Middle Temporal Gyrus' | -49 | -42 | 1 |
|  | Default mode | 'Inferior Frontal Gyrus' | -46 | 31 | -13 |
|  | Default mode | 'Inferior Frontal Gyrus' | 49 | 35 | -12 |
|  | | | | | |
| Cognitive | Memory retrieval | 'Cingulate Gyrus' | -2 | -35 | 31 |
|  | Memory retrieval | 'Precuneus' | -7 | -71 | 42 |
|  | Memory retrieval | 'Precuneus' | 11 | -66 | 42 |
|  | Memory retrieval | 'Precuneus' | 4 | -48 | 51 |
|  | Memory retrieval | 'Cingulate Gyrus' | 2 | -24 | 30 |
|  | | | | | |
| Sensory | Visual | 'Parahippocampa Gyrus' | 18 | -47 | -10 |
|  | Visual | 'Middle Temporal Gyrus' | 40 | -72 | 14 |
|  | Visual | 'Cuneus' | 8 | -72 | 11 |
|  | Visual | 'Cuneus' | -8 | -81 | 7 |
|  | Visual | 'Sub-Gyral' | -28 | -79 | 19 |
|  | Visual | 'Lingual Gyrus' | 20 | -66 | 2 |
|  | Visual | 'Cuneus' | -24 | -91 | 19 |
|  | Visual | 'Lingual Gyrus' | 27 | -59 | -9 |
|  | Visual | 'Lingual Gyrus' | -15 | -72 | -8 |
|  | Visual | 'Cuneus' | -18 | -68 | 5 |
|  | Visual | 'Inferior Occipital Gyrus' | 43 | -78 | -12 |
|  | Visual | 'Middle Occipital Gyrus' | -47 | -76 | -10 |
|  | Visual | 'Cuneus' | -14 | -91 | 31 |
|  | Visual | 'Precuneus' | 15 | -87 | 37 |
|  | Visual | 'Sub-Gyral' | 29 | -77 | 25 |
|  | Visual | 'Lingual Gyrus' | 20 | -86 | -2 |
|  | Visual | 'Cuneus' | 15 | -77 | 31 |
|  | Visual | 'Lingual Gyrus' | -16 | -52 | -1 |
|  | Visual | 'Middle Occipital Gyrus' | 42 | -66 | -8 |
|  | Visual | 'Cuneus' | 24 | -87 | 24 |
|  | Visual | 'Precuneus' | 6 | -72 | 24 |
|  | Visual | 'Middle Occipital Gyrus' | -42 | -74 | 0 |
|  | Visual | 'Lingual Gyrus' | 26 | -79 | -16 |
|  | Visual | 'Precuneus' | -16 | -77 | 34 |
|  | Visual | 'Cuneus' | -3 | -81 | 21 |
|  | Visual | 'Inferior Occipital Gyrus' | -40 | -88 | -6 |
|  | Visual | 'Middle Occipital Gyrus' | 37 | -84 | 13 |
|  | Visual | 'Cuneus' | 6 | -81 | 6 |
|  | Visual | 'Middle Occipital Gyrus' | -26 | -90 | 3 |
|  | Visual | 'Middle Occipital Gyrus' | -33 | -79 | -13 |
|  | Visual | 'Middle Occipital Gyrus' | 37 | -81 | 1 |
|  | | | | | |
| Cognitive | Fronto-parietal Task Control | 'Middle Frontal Gyrus' | -44 | 2 | 46 |
|  | Fronto-parietal Task Control | 'Middle Frontal Gyrus' | 48 | 25 | 27 |
|  | Fronto-parietal Task Control | 'Inferior Frontal Gyrus' | -47 | 11 | 23 |
|  | Fronto-parietal Task Control | 'Inferior Parietal Lobule' | -53 | -49 | 43 |
|  | Fronto-parietal Task Control | 'Superior Frontal Gyrus' | -54 | 11 | 64 |
|  | Fronto-parietal Task Control | 'Middle Temporal Gyrus' | -66 | -53 | -14 |
|  | Fronto-parietal Task Control | 'Superior Frontal Gyrus' | -78 | 45 | -15 |
|  | Fronto-parietal Task Control | 'Middle Frontal Gyrus' | -90 | 54 | -13 |
|  | Fronto-parietal Task Control | 'Middle Frontal Gyrus' | -102 | 10 | 33 |
|  | Fronto-parietal Task Control | 'Inferior Frontal Gyrus' | -41 | 6 | 33 |
|  | Fronto-parietal Task Control | 'Middle Frontal Gyrus' | -42 | 38 | 21 |
|  | Fronto-parietal Task Control | 'Middle Frontal Gyrus' | 38 | 43 | 15 |
|  | Fronto-parietal Task Control | 'Inferior Parietal Lobule' | 49 | -42 | 45 |
|  | Fronto-parietal Task Control | 'Superior Parietal Lobule' | -28 | -58 | 48 |
|  | Fronto-parietal Task Control | 'Inferior Parietal Lobule' | 44 | -53 | 47 |
|  | Fronto-parietal Task Control | 'Superior Frontal Gyrus' | 32 | 14 | 56 |
|  | Fronto-parietal Task Control | 'Inferior Parietal Lobule' | 37 | -65 | 40 |
|  | Fronto-parietal Task Control | 'Inferior Parietal Lobule' | -42 | -55 | 45 |
|  | Fronto-parietal Task Control | 'Middle Frontal Gyrus' | 40 | 18 | 40 |
|  | Fronto-parietal Task Control | 'Middle Frontal Gyrus' | -34 | 55 | 4 |
|  | Fronto-parietal Task Control | 'Middle Frontal Gyrus' | -42 | 45 | -2 |
|  | Fronto-parietal Task Control | 'Inferior Parietal Lobule' | 33 | -53 | 44 |
|  | Fronto-parietal Task Control | 'Middle Frontal Gyrus' | 43 | 49 | -2 |
|  | Fronto-parietal Task Control | 'Middle Frontal Gyrus' | -42 | 25 | 30 |
|  | Fronto-parietal Task Control | 'Medial Frontal Gyrus' | -3 | 26 | 44 |
|  | | | | | |
| Cognitive | Salience | 'Paracentral Lobule' | 11 | -39 | 50 |
|  | Salience | 'Supramarginal Gyrus' | 55 | -45 | 37 |
|  | Salience | 'Middle Frontal Gyrus' | 42 | 0 | 47 |
|  | Salience | 'Sub-Gyral' | 31 | 33 | 26 |
|  | Salience | 'Inferior Frontal Gyrus' | 48 | 22 | 10 |
|  | Salience | 'Extra-Nuclear' | -35 | 20 | 0 |
|  | Salience | 'Insula' | 36 | 22 | 3 |
|  | Salience | 'Inferior Frontal Gyrus' | 37 | 32 | -2 |
|  | Salience | 'Extra-Nuclear' | 34 | 16 | -8 |
|  | Salience | 'Anterior Cingulate' | -11 | 26 | 25 |
|  | Salience | 'Cingulate Gyrus' | -1 | 15 | 44 |
|  | Salience | 'Middle Frontal Gyrus' | -28 | 52 | 21 |
|  | Salience | 'undefined' | 0 | 30 | 27 |
|  | Salience | 'Cingulate Gyrus' | 5 | 23 | 37 |
|  | Salience | 'Anterior Cingulate' | 10 | 22 | 27 |
|  | Salience | 'Middle Frontal Gyrus' | 31 | 56 | 14 |
|  | Salience | 'Superior Frontal Gyrus' | 26 | 50 | 27 |
|  | Salience | 'Superior Frontal Gyrus' | -39 | 51 | 17 |
|  | | | | | |
| Sensory (limbic) | Subcortical | 'Extra-Nuclear' | 6 | -24 | 0 |
|  | Subcortical | 'Extra-Nuclear' | -2 | -13 | 12 |
|  | Subcortical | 'Thalamus' | -10 | -18 | 7 |
|  | Subcortical | 'Thalamus' | 12 | -17 | 8 |
|  | Subcortical | 'undefined' | -5 | -28 | -4 |
|  | Subcortical | 'Lentiform Nucleus' | -22 | 7 | -5 |
|  | Subcortical | 'Lentiform Nucleus' | -15 | 4 | 8 |
|  | Subcortical | 'Extra-Nuclear' | 31 | -14 | 2 |
|  | Subcortical | 'Lentiform Nucleus' | 23 | 10 | 1 |
|  | Subcortical | 'Extra-Nuclear' | 29 | 1 | 4 |
|  | Subcortical | 'Extra-Nuclear' | -31 | -11 | 0 |
|  | Subcortical | 'Extra-Nuclear' | 15 | 5 | 7 |
|  | Subcortical | 'Thalamus' | 9 | -4 | 6 |
|  | | | | | |
| Cognitive | Ventral attention | 'Superior Frontal Gyrus' | -10 | 11 | 67 |
|  | Ventral attention | 'Inferior Parietal Lobule' | 54 | -43 | 22 |
|  | Ventral attention | 'Superior Temporal Gyrus' | -56 | -50 | 10 |
|  | Ventral attention | 'Superior Temporal Gyrus' | -55 | -40 | 14 |
|  | Ventral attention | 'Superior Temporal Gyrus' | 52 | -33 | 8 |
|  | Ventral attention | 'Middle Temporal Gyrus' | 51 | -29 | -4 |
|  | Ventral attention | 'Superior Temporal Gyrus' | 56 | -46 | 11 |
|  | Ventral attention | 'Inferior Frontal Gyrus' | 53 | 33 | 1 |
|  | Ventral attention | 'Inferior Frontal Gyrus' | -49 | 25 | -1 |
|  | | | | | |
| Cognitive | Dorsal attention | 'Precuneus' | 10 | -62 | 61 |
|  | Dorsal attention | 'Middle Temporal Gyrus' | -52 | -63 | 5 |
|  | Dorsal attention | 'Precuneus' | 22 | -65 | 48 |
|  | Dorsal attention | 'Middle Temporal Gyrus' | 46 | -59 | 4 |
|  | Dorsal attention | 'Superior Parietal Lobule' | 25 | -58 | 60 |
|  | Dorsal attention | 'Sub-Gyral' | -33 | -46 | 47 |
|  | Dorsal attention | 'Precuneus' | -27 | -71 | 37 |
|  | Dorsal attention | 'Middle Frontal Gyrus' | -32 | -1 | 54 |
|  | Dorsal attention | 'Sub-Gyral' | -42 | -60 | -9 |
|  | Dorsal attention | 'Superior Parietal Lobule' | -17 | -59 | 64 |
|  | Dorsal attention | 'Middle Frontal Gyrus' | 29 | -5 | 54 |
|  | | | | | |
| Cognitive | Cerebellar | 'Declive' | -16 | -65 | -20 |
|  | Cerebellar | 'Culmen' | -32 | -55 | -25 |
|  | Cerebellar | 'Declive' | 22 | -58 | -23 |
|  | Cerebellar | 'Declive' | 1 | -62 | -18 |
|  | | | | | |
| Not defined. Associated with limbic processing | Uncertain | 'Lingual Gyrus' | -25 | -98 | -12 |
|  | Uncertain | 'Inferior Occipital Gyrus' | 27 | -97 | -13 |
|  | Uncertain | 'Middle Frontal Gyrus' | 24 | 32 | -18 |
|  | Uncertain | 'Inferior Temporal Gyrus' | -56 | -45 | -24 |
|  | Uncertain | 'Sub-Gyral' | 8 | 41 | -24 |
|  | Uncertain | 'Parahippocampa Gyrus' | -21 | -22 | -20 |
|  | Uncertain | 'Culmen' | 17 | -28 | -17 |
|  | Uncertain | 'Parahippocampa Gyrus' | -37 | -29 | -26 |
|  | Uncertain | 'undefined' | 65 | -24 | -19 |
|  | Uncertain | 'Fusiform Gyrus' | 52 | -34 | -27 |
|  | Uncertain | 'Middle Temporal Gyrus' | 55 | -31 | -17 |
|  | Uncertain | 'Middle Frontal Gyrus' | 34 | 38 | -12 |
|  | Uncertain | 'Middle Temporal Gyrus' | -58 | -26 | -15 |
|  | Uncertain | 'Inferior Frontal Gyrus' | 27 | 16 | -17 |
|  | Uncertain | 'Inferior Frontal Gyrus' | -31 | 19 | -19 |
|  | Uncertain | 'Lingual Gyrus' | 8 | -91 | -7 |
|  | Uncertain | 'Inferior Occipital Gyrus' | 17 | -91 | -14 |
|  | Uncertain | 'Lingual Gyrus' | -12 | -95 | -13 |
|  | Uncertain | 'Superior Frontal Gyrus' | -21 | 41 | -20 |
|  | Uncertain | 'Declive' | -18 | -76 | -24 |
|  | Uncertain | 'Uvula' | 17 | -80 | -34 |
|  | Uncertain | 'Tuber' | 35 | -67 | -34 |
|  | Uncertain | 'Uncus' | 33 | -12 | -34 |
|  | Uncertain | 'Uncus' | -31 | -10 | -36 |
|  | Uncertain | 'Inferior Temporal Gyrus' | 49 | -3 | -38 |
|  | Uncertain | 'Inferior Temporal Gyrus' | -50 | -7 | -39 |
|  | Uncertain | 'Fusiform Gyrus' | -47 | -51 | -21 |
|  | Uncertain | 'Fusiform Gyrus' | 46 | -47 | -17 |

**Supplemental Table 1**. Per our hypotheses Power’s brain networks ^2^ were classified into cognitive, sensory, and limbic networks (1^st^ and 2^nd^ columns).

**References related to the supplementary data**

1. Finn ES, Shen X, Scheinost D, et al. Functional connectome fingerprinting: identifying individuals using patterns of brain connectivity. *Nature neuroscience.* 2015;18(11):1664-1671.

2. Power JD, Cohen AL, Nelson SM, et al. Functional network organization of the human brain. *Neuron.* 2011;72(4):665-678.
